# Translation of a Human‐Based Malaria‐on‐a‐Chip Phenotypic Disease Model for In Vivo Applications

**DOI:** 10.1002/advs.202505206

**Published:** 2025-07-21

**Authors:** Michael J. Rupar, Hannah M. Hanson, Brianna L. Botlick, Narasimhan Sriram, Stephanie Rogers, Justin Zuniga, Zhanhe Liu, Steven J. Trimmer, Joseph M. Ciurca, Christopher J. Long, Christopher W. McAleer, Stephan Schmidt, Paola Favuzza, Philip Lowe, Nathalie Gobeau, James J. Hickman

**Affiliations:** ^1^ Hesperos, Inc. 12501 Research Parkway, Suite 100 Orlando FL 32826 USA; ^2^ Department of Chemistry University of Central Florida Orlando Florida 32826 USA; ^3^ Department of Pharmaceutics Center for Pharmacometrics and Systems Pharmacology College of Pharmacy University of Florida Orlando Florida 32827 USA; ^4^ Medicines for Malaria Ventures Rte de Pré‐Bois 20 Meyrin 1215 Switzerland

**Keywords:** malaria, microphysiological systems, organ‐on‐a‐chip, Plasmodium falciparum

## Abstract

In 2023 malaria claimed ≈600000 lives, with 90% of those deaths attributed to the *Plasmodium falciparum* parasite. This resurgence in mortality emphasizes the necessity of adopting alternative models to accelerate therapeutic development. The Malaria‐on‐a‐Chip model used here incorporated human liver, spleen, and endothelium with *P. falciparum‐*infected blood, and was maintained for 7 days using serum‐free medium. This model sustained all stages of the intraerythrocytic life cycle and allowed for organ–organ interaction, providing advantageous preclinical insight into malaria pathophysiology. Chloroquine, lumefantrine, or artesunate were delivered as monotherapies to 3D7 or W2‐infected systems. Dose‐dependent parasite clearance was observed in both strains for all compounds. Recrudescence occurred in the 3D7‐infected model following treatment with chloroquine or lumefantrine, but not artesunate. In W2‐infected systems, chloroquine and lumefantrine treatment resulted in parasitemia stabilization by day 7, while artesunate further reduced parasitemia. Population dynamics modeling of pharmacokinetic and pharmacodynamic (PK/PD) outcomes were utilized to predict human in vivo parameters for efficacy and off‐target toxicity using in vitro results.

## Graphical abstract

Inoculation of the Malaria‐on‐a‐Chip model with human erythrocytes infected with *Plasmodium falciparum* can support the entire intraerythrocytic lifecycle for 7 days in vitro. Utilizing this human‐based, serum‐free model provides an alternative to animal testing by introducing a human‐based, preclinical alternative for antimalarial therapeutic delivery. This digital twin enables the translation of in vitro PK/PD outcomes to in vivo studies.

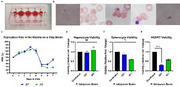

## 1 Introduction

The deadliest strain of the malaria‐causing *Plasmodium* genus is the *falciparum* species; as of 2024, this parasite is estimated to have caused malaria infection in more than 250 million people each year, leading to more than 600000 deaths annually. Malaria primarily burdens China, Africa, and Central and South America, and especially devastates vulnerable populations such as pregnant women and children.^[^ ^1^ ^]^ The most severe effects are seen in Africa, where *P. falciparum* is highly prevalent, comprising 95% of recorded malaria fatalities. Despite ongoing mitigation efforts, repeated emergence of chemo‐resistant *P. falciparum* strains has enabled the parasite to continually evade eradication. Parasite strains resistant to both preventative therapies and post‐infection treatment options have surfaced.^[^ ^2^ ^]^ These resistant strains are theorized to have contributed to a recent resurgence in cases; according to the World Health Organization, the estimated number of malaria cases has steadily increased since 2020, deviating from the stable case estimations of years prior. This alarming trend has solidified malaria as a persistent global health burden. This has also highlighted the necessity of pursuing all available avenues for modeling disease and drug efficacy, to accelerate availability of novel treatment options. Adoption of alternative, in vitro models may improve success in the development of new therapeutics. These models may reduce the drug‐development timeline by providing insight into the effects of compounds on human physiology in a well‐defined and scalable microenvironment. The Malaria‐on‐a‐Chip model in this study, composed of primary human liver, spleen, endothelium, and blood, seeks to provide new insights into pre‐clinical drug efficacy and safety as well as establish digital twins to link the in vitro and in vivo results.

The malaria parasite is transmitted to humans when a female *Anopheles* mosquito infected with *P. falciparum* partakes in a blood meal. Invading sporozoites first migrate to the liver to incubate and produce merozoites, which enter the bloodstream after about 7 days to invade healthy erythrocytes.^[^ ^1^ ^]^ This blood‐stage of infection is known as the asexual intraerythrocytic lifecycle, which is the focus of this study. *P. falciparum* causes severe disease in the human host due in part to the ability of the merozoite progeny to infect a high proportion of red blood cells (RBCs).^[^ ^3^ ^]^ After invasion, merozoites progress through the ring, trophozoite, and schizont stages, filling the erythrocyte and eventually causing it to burst. Each burst red blood cell releases multiple merozoites, increasing the infection rate and accelerating disease progression.^[^ ^4^ ^]^

A characteristic element of *P. falciparum* pathophysiology is the sequestration of infected red blood cells (iRBCs) in the endothelium of the microvasculature. Human Umbilical Vein Endothelial Cells (HUVECs) were used in this model to reflect this disease pathology. Sequestration of iRBCs occurs when infected erythrocytes containing mature parasites adhere to the endothelium.^[^ ^5^ ^]^ This sequestration, also referred to as endothelial cytoadherence, enables the parasite to avoid clearance by the organ responsible for filtering the blood cell population – the spleen.^[^ ^6^ ^]^

In vivo, the spleen is integral for parasite clearance, employing both mechanical features and cytokine chemotaxis. iRBCs lose deformability after parasite invasion, resulting in an inability to navigate through the interendothelial slits within the marginal zone of the spleen.^[^ ^7^ ^]^ After being slowed and stopped by the narrow passes of the slits, the infected cells are targeted for phagocytosis by resident macrophages.^[^ ^8^ ^]^ Immune responses are generated in the white pulp of the spleen to further assist in clearing infection. To recapitulate these functions, primary human splenocytes were included in this model.

In addition to these two organ constructs, hepatocytes were also incorporated into this phenotypic disease model. Although hepatic cells are involved in the pre‐erythrocytic phase of infection,^[^ ^9^ ^]^ they were included in the Malaria‐on‐a‐Chip model to simulate the first‐pass metabolism of commonly used antimalarials in vitro. Three approved antimalarial therapeutics were administered: chloroquine, artesunate, and lumefantrine. To model drug resistance, and to address the growing concern of this phenomenon in the development of novel therapeutics, systems were infected with either the chloroquine‐sensitive 3D7 strain of the parasite, or the chloroquine‐resistant W2 strain.^[^ ^10^ ^]^

Existing pharmacokinetic‐pharmacodynamic (PK/PD) modeling for anti‐malarial compounds has focused on optimizing dosing regimens for existing compounds in several applications, including for effectiveness and ease of adherence to the regimen. PK/PD modeling has shown great promise for adjusting dosing for populations with specific restrictions or complications, such as pregnant women, children, those with malnutrition, and patients with existing immunodeficiencies. PK/PD techniques enable small‐scale studies on human patients to distinguish effects and prepare useful adjustments to treatment regimens. The PK/PD models can be used to generate digital medical twins, which are digital representations that combine models of human biology with operational data to mimic the structure, context, and behavior of medical or biomedical systems. The use of a population‐PK approach with PK/PD modeling enables the production of digital medical twins for the Malaria‐on‐a‐Chip systems, which can help inform outcomes for specific patient populations. The major benefit in clinical PK/PD applications is that PK/PD modeling can clarify relationships between drug exposures and key patient outcomes.^[^ ^11^ ^]^ However, this approach requires clinical data from human patients as the baseline from which to make adjustments and adaptations to treatment options, and while clinical digital twins are possible with this data, their predictability is not ideal.

This study utilized the previously established Malaria‐on‐a‐Chip model^[^ ^12^ ^]^ to more thoroughly evaluate the parasitic intraerythrocytic life cycle and monitor effects of infection on cell health and immune response. Following administration of treatment, this study monitored pharmacodynamic effects of the antimalarials used, the pharmacokinetic outcomes following liver metabolism, drug efficacy in parasite clearance and recrudescence, as well as immune response to both infection and treatment. These observations led to the generation of clinically relevant PK/PD models. Thus, this model demonstrates potential future use for the development of a microphysiological system digital twin to inform the digital representations for clinical predictability of no adverse effect levels (NOAEL), maximum tolerable doses (MTD), and half‐maximal effect concentrations (EC_50_) for existing and new therapeutics.

## 2 Results

### 2.1 Characterization of the Malaria‐on‐a‐Chip Phenotypic Disease Model

The previously established Malaria‐on‐a‐Chip model^[^ ^12^ ^]^ was enhanced for this study. The schematics (Figure S1, Supporting Information) remain the same, however the platform was further optimized by modifying the medium formulation to generate more robust cell viability. No significant changes in hepatocyte viability were observed but spleen and endothelium viability were significantly improved. No significant changes in parasite replication rates were observed. The optimized organ constructs in this platform were still interconnected in a serum‐free, pumpless, microfluidic design. Internal compartments were constructed in a way that allows for gravity‐driven, physiological flow with minimal shear stress allowing for even distribution of medium throughout the system. The bidirectional flow generated even mixing throughout the system by 24 h to enable organ to organ interaction, delivery of therapeutic compounds, and recirculation of free‐floating erythrocytes.

**Figure 1:**
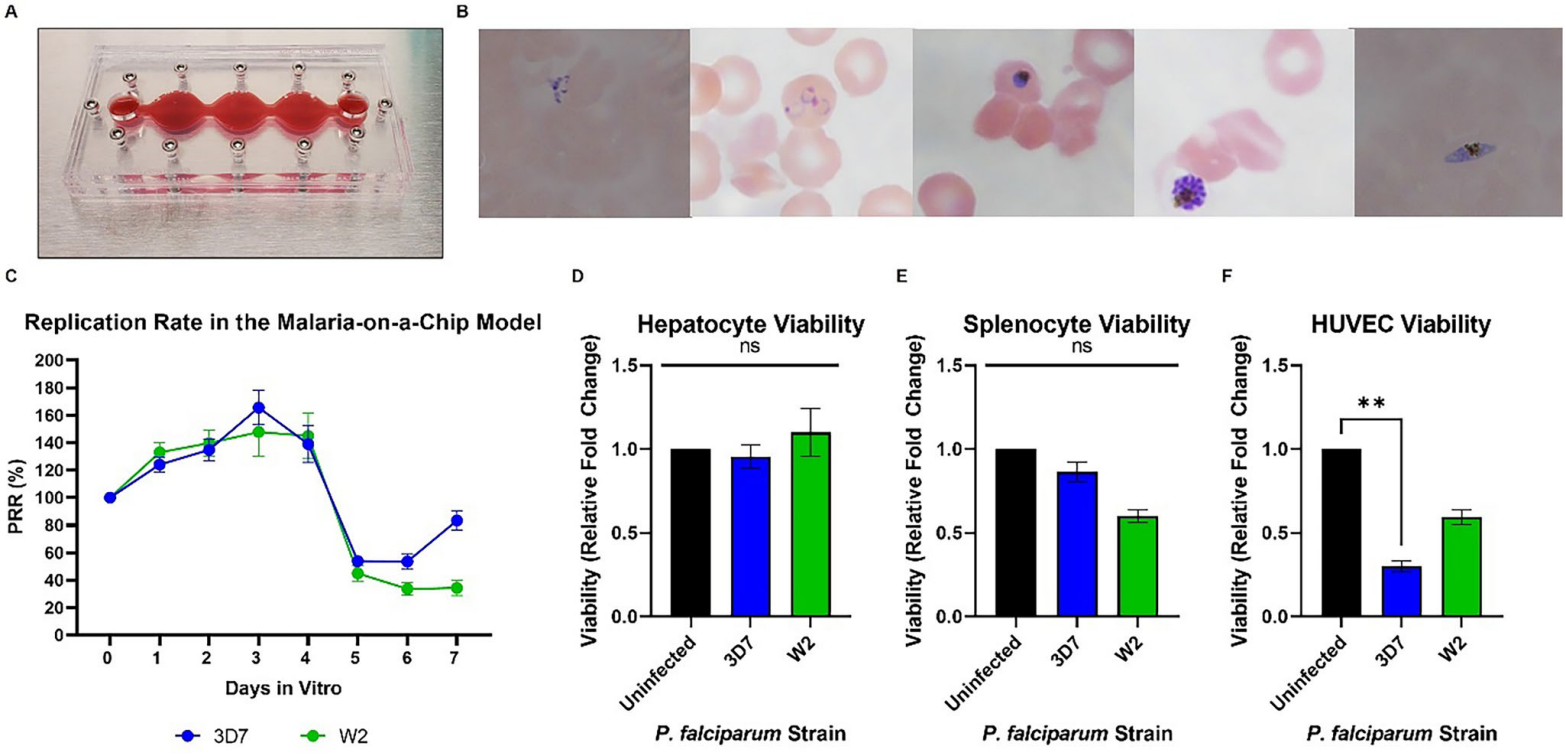
Malaria‐on‐a‐Chip Model. A) Multiorgan systems were assembled with hepatocytes, splenocytes, and HUVECS, and then RBCs were introduced to the model. RBCs were either healthy, uninfected cells or infected with the 3D7 or W2 strain of P. falciparum. B) Blood samples were collected from the system for Giemsa staining to visualize the parasite lifecycle within the model. Every stage of the intraerythrocytic parasite was observed (pictured from left to right: merozoites, ring stage, immature and mature schizonts, and gametocytes) C) Parasitemia levels were monitored daily over the course of 7 days to determine the replication rate of each strain within the model. D–F) Viability studies were conducted for the liver, spleen, and endothelium on day 7 immediately following the disassembly of the multi‐organ systems. No significant differences in hepatocyte or splenocyte viability were observed when exposed to infection. Decline in HUVEC viability did occur with both strains, however only infection with the 3D7 strain resulted in a significant decrease in viability. Data was analyzed via One‐way ANOVA; *n* = 35; Mean ± SEM.

The Malaria‐on‐a‐Chip system (**Figure** **1**) was maintained for 7 days, and blood samples were collected for observation of the erythrocytic life cycle in the model (Figure 1B) as well as quantification of the parasite to establish replication rates within the system (Figure 1C) for each strain. A lag in the proliferation rate of both strains was observed. This is likely due to the atmospheric conditions in which the systems were maintained. *P. falciparum* has an affinity for hypoxic culture conditions (5% CO2, 5% O2, 90% N2), yet the Malaria‐on‐a‐Chip platform was maintained in standard incubator conditions (5% CO2, 21% O2, 74% N2). This increase in oxygen concentration slows the proliferation rate of the parasite.

All stages of the erythrocytic life cycle were observed, including the occasional sexual gametocyte. In the systems infected with the 3D7 strain (*n* = 35), a near doubling of the population was observed by day 3 reaching ≈6%. For the systems inoculated with the W2 strain (*n* = 30), a slightly lower replication rate was observed. The highest parasitemia observed in a single W2 infected system was ≈10%, while the highest parasitemia on average of ≈5% was observed on day 3.

Upon disassembly, viability studies for organ constructs maintained in each infected strain culture were compared to those systems which were uninfected (Figure 1D–F). No significant differences were determined in the liver organ construct. While not statistically significant, slight decreases in splenic organ viability were observed in both strains. The viability of the endothelium organ construct was reduced when infected with either strain, but only significantly reduced in the 3D7‐infected systems.

### 2.2 Antimalarial Efficacy in the Malaria‐on‐a‐Chip Model

**Figure 2:**
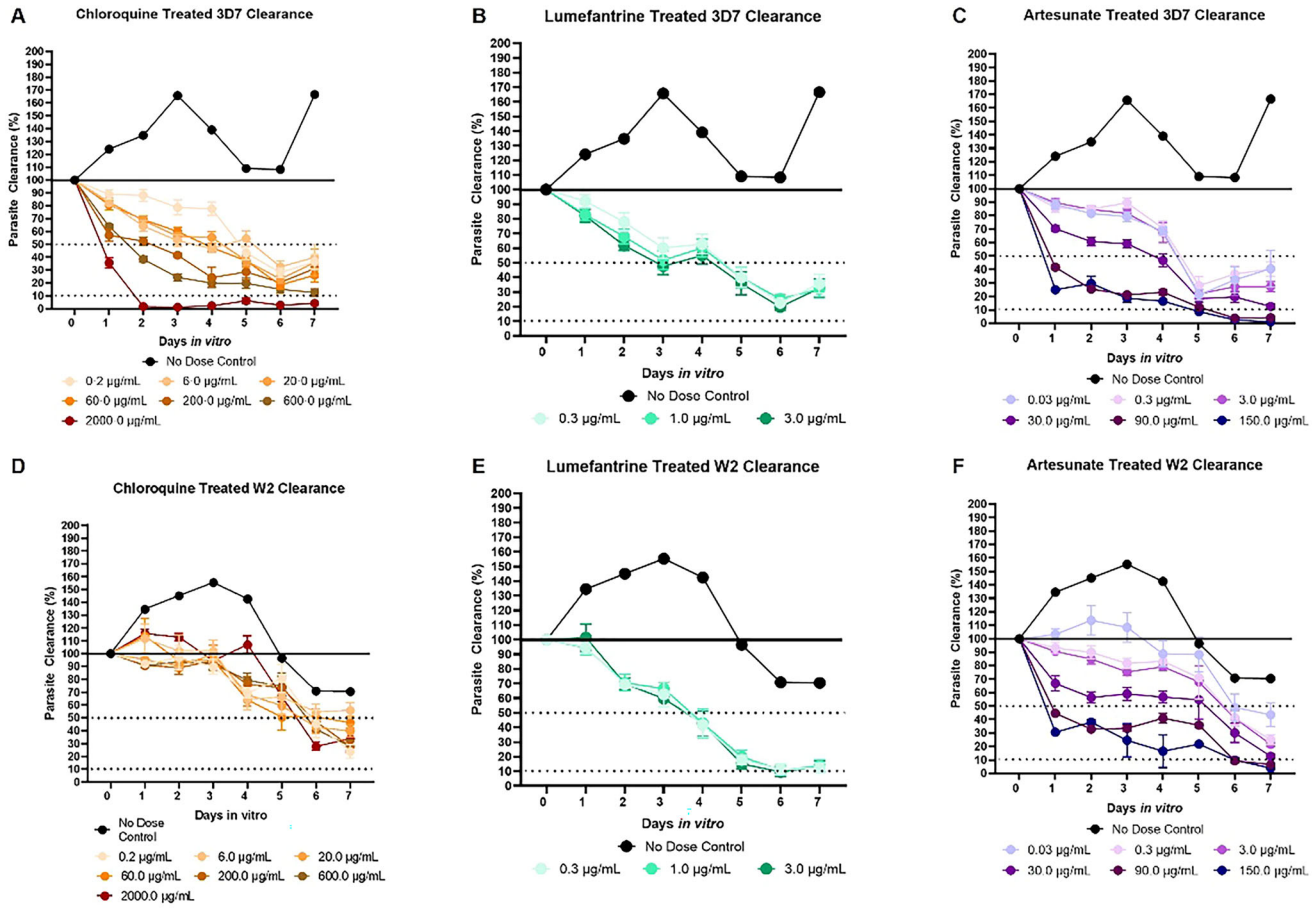
Antimalarial Effects on Parasite Clearance in the Malaria‐on‐a‐Chip Model. Following treatment, parasitemia was monitored for both strains over 7 days. In the chloroquine‐treated systems A,D), notable differences were observed between the 3D7 strain and the W2 strain. 3D7 was cleared by chloroquine in a dose‐dependent manner, while the W2 systems had similar clearance rates across doses and failed to drop below 50% clearance within the first 4 days. In the lumefantrine‐treated systems B,E), no significant variations were observed across treatment groups for either strain. In the artesunate‐treated systems C,F), a dose‐dependent effect was observed for both strains. 3D7 showed a higher initial susceptibility to artesunate treatment than W2, although 3D7 did observe a higher recrudescence by day 7 compared to W2.

In the 3D7‐infected systems treated with chloroquine (**Figure** **2**A), a single administered dose of 200.0 µg mL^−1^ or higher was necessary to reduce the parasitemia by 50% before day 4. Recrudescence was observed in all doses excluding the 600.0 and 2000.0 µg mL^−1^ doses. In the 3D7‐infected systems treated with lumefantrine (Figure 2B), the administered 3.0 µg mL^−1^ dose reached 50% clearance by day 3. However, recrudescence was observed in all doses by day 4. In the 3D7‐infected systems treated with artesunate (Figure 2C), a single administered dose of 30.0 µg mL^−1^ or higher was necessary to reduce parasitemia by 50% before day 4. Furthermore, in doses of 30.0 µg mL^−1^ and higher, no recrudescence was observed.

In the W2‐infected systems treated with chloroquine (Figure 2D), no clear differences between treatment groups were observed in the first 4 days, and all concentrations failed to clear more than 50% of the parasite before blood addition on day 4. In the W2‐infected systems treated with lumefantrine (Figure 2E), a single dose of 0.3 µg mL^−1^ and higher reached 50% clearance by day 4. A slight recrudescence was observed in all doses by day 7. In the W2‐infected systems treated with artesunate (Figure 2F), a single administered dose of 90.0 µg mL^−1^ or higher was necessary to reduce the parasitemia by 50% before day 4. Parasitemia for all the administered doses continued to decline over the 7 days, and no recrudescence was observed.

### 2.3 Antimalarial Off‐Target Toxicity in the Malaria‐on‐a‐Chip Model

**Figure 3:**
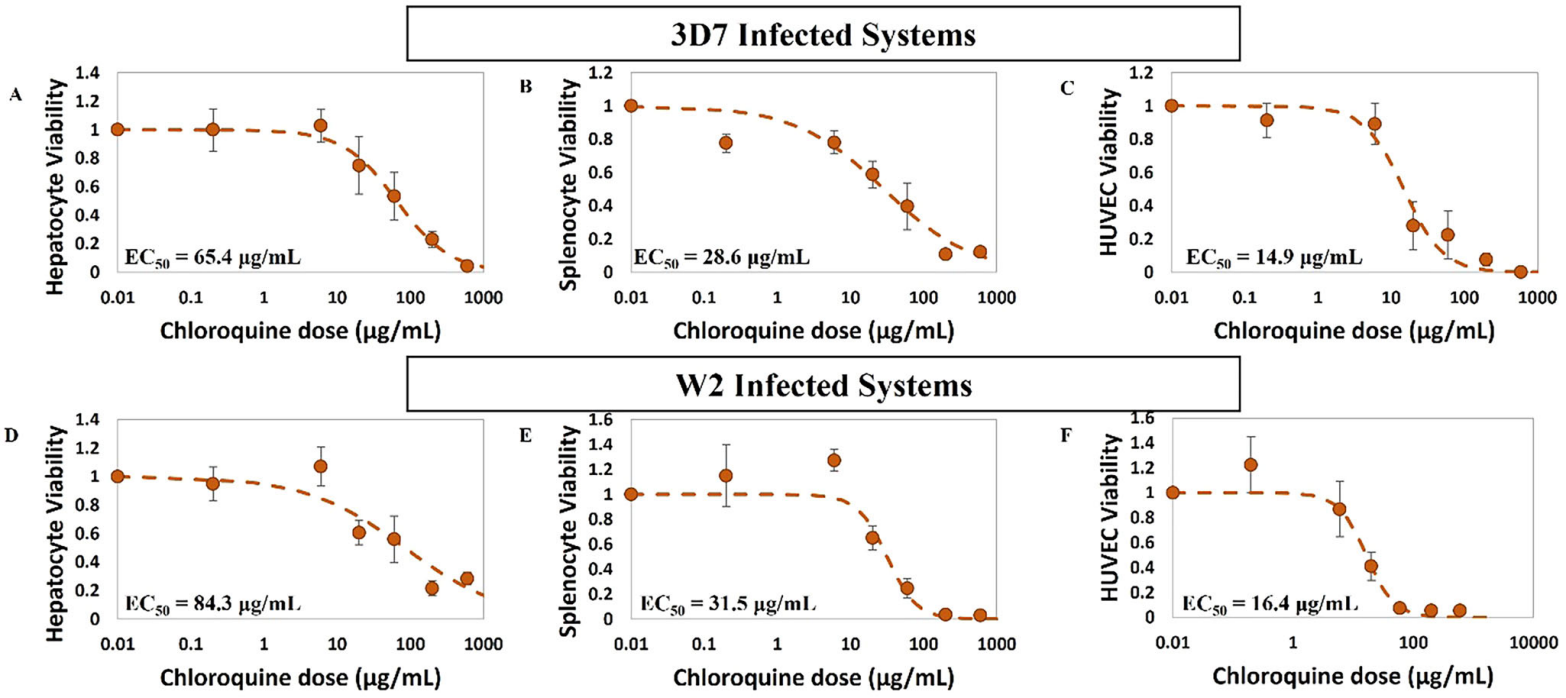
Dose‐response of organ viability changes to chloroquine in the Malaria‐on‐a‐Chip Model. Dose‐dependent changes in hepatocyte, splenocyte, and HUVEC viability were characterized for EC_50_ using non‐linear regression analysis A–C). For the 3D7‐infected systems, the HUVECs had the lowest EC_50_ with respect to chloroquine, while splenocytes and hepatocytes exhibited slightly less susceptibility to chloroquine‐induced viability changes (EC_50_ of 14.9, 28.6, and 65.4 µg mL^−1^, respectively. D–F), W2‐infected systems showed similar organ toxicity as 3D7 systems, with the HUVECs, splenocytes, and hepatocytes demonstrating EC_50_ values of 16.4, 31.5, and 84.3 µg mL^−1^, respectively.

**Figure 4:**
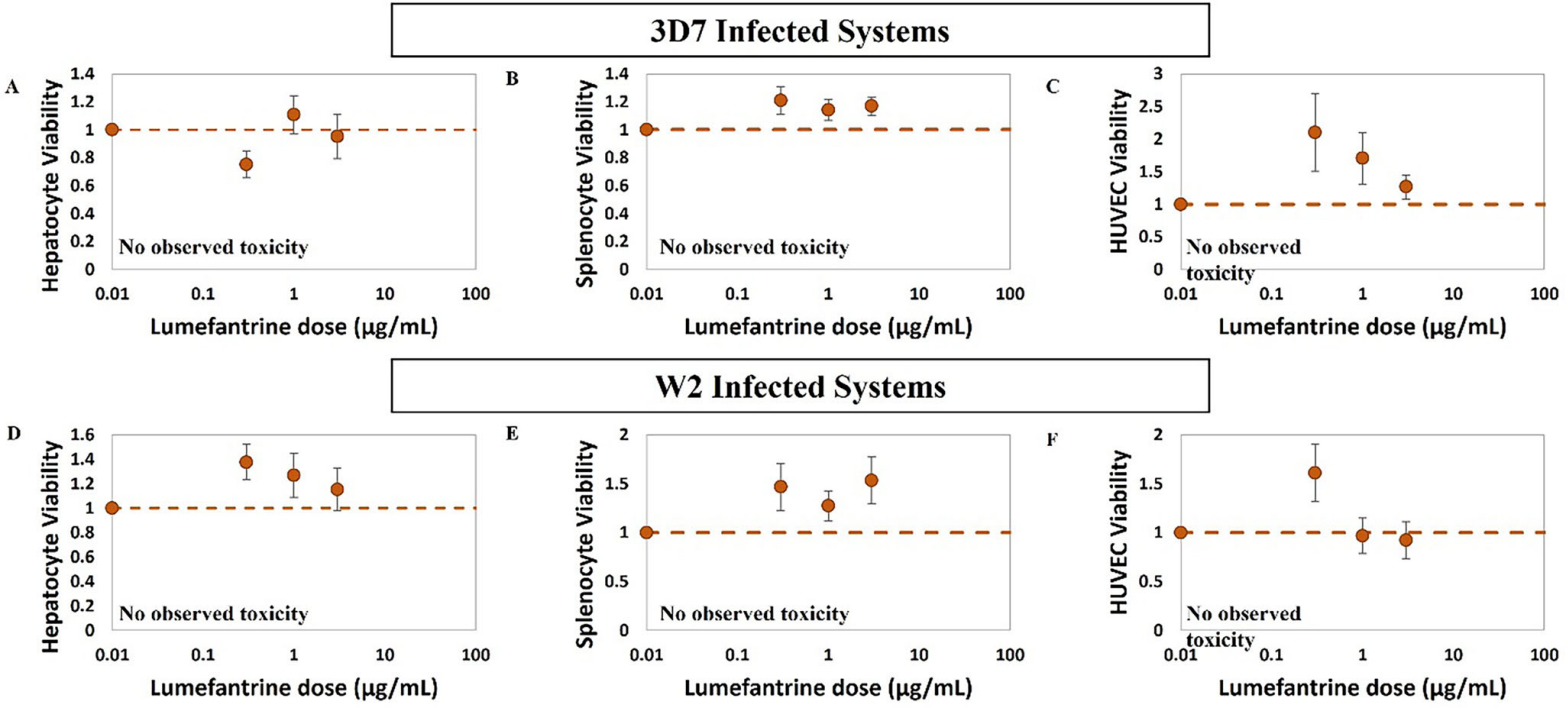
Dose‐response of organ viability changes to lumefantrine in the Malaria‐on‐a‐Chip Model. Dose‐dependent changes in hepatocyte, splenocyte, and HUVEC viability were characterized for EC_50_ using non‐linear regression analysis A–C). For the 3D7‐infected systems and D–F) for W2‐infected systems, no lumefantrine‐induced viability changes were observed for hepatocytes, splenocytes, or HUVECs within the range tested.

**Figure 5:**
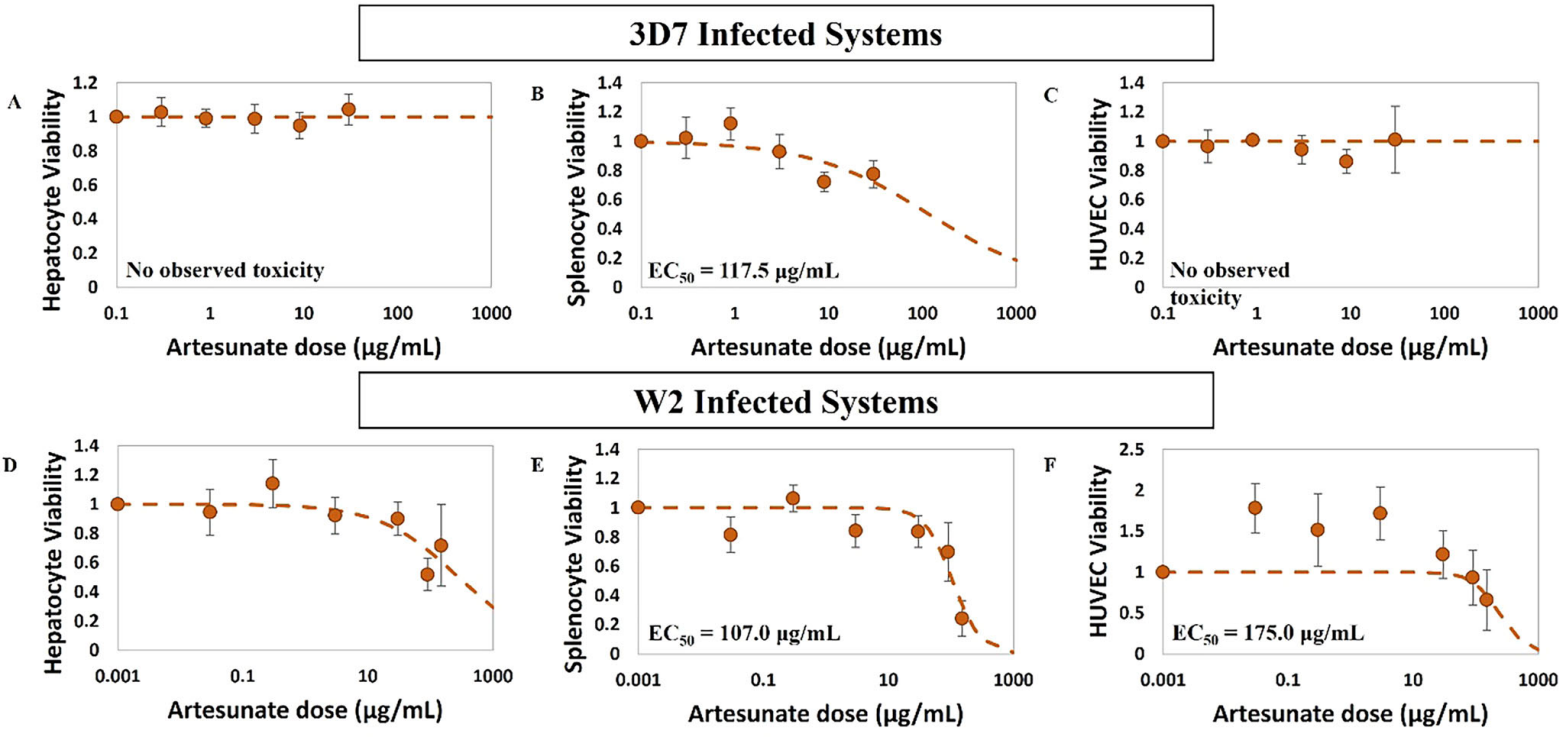
Dose‐response of organ viability changes to artesunate in the Malaria‐on‐a‐Chip Model. Dose‐dependent changes in hepatocyte, splenocyte, and HUVEC viability were characterized for EC_50_ using non‐linear regression analysis A–C). For the 3D7‐infected systems, only the splenocytes demonstrated artesunate‐induced viability changes, with an estimated EC_50_ above the highest dose tested. D–F), W2‐infected systems exhibited changes in viability with respect to artesunate dose for all organs, though only at the highest concentrations tested, with the HUVECs, splenocytes, and hepatocytes demonstrating EC50 values of 175, 107, and 280 µg mL^−1^, respectively.

Non‐linear regression analysis was performed to determine the dependence of drug dose on organ viability in the Malaria‐on‐a‐Chip model, characterized chiefly by the effective concentration causing a 50% loss in viability (EC_50_) (Figures 3, 4, 5). The EC_50_ values were similar between 3D7 and W2‐infected systems for each organ type. HUVECs demonstrated the most susceptibility to chloroquine, with EC_50_ of 14.9 and 16.4 µg mL^−1^ for 3D7 and W2‐infected systems, respectively. The splenocytes and hepatocytes demonstrated slightly higher EC_50_ values for chloroquine than the HUVECs, with average EC_50_ values of 30.0 and 74.5 µg mL^−1^, respectively (**Figure** **3**). As no toxicity was observed in the organs dosed with lumefantrine, regression analysis did not result in an EC_50_ value for any of the organs, for either the 3D7 or W2‐infected systems (**Figure** **4**). Artesunate dosing resulted in EC_50_ values above 100.0 µg mL^−1^ for the splenocytes and above the highest concentrations tested for the hepatocytes and HUVECs (**Figure** **5**).

### 2.4 Development of Immune Response Profiles using the Malaria‐on‐a‐Chip Model

**Figure 6:**
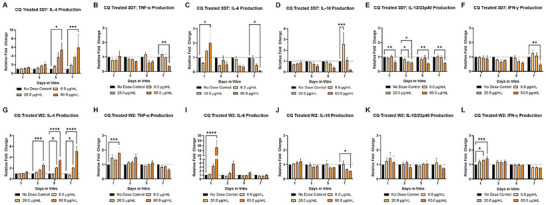
Chloroquine Effects on Cytokine Production in the Malaria‐on‐a‐Chip Model. Medium collections were performed on days 1, 3, 5, and 7 for monitoring the release of proinflammatory cytokines (TNF‐α, IL‐6, IL‐12, and INF‐γ) and regulatory cytokines (IL‐4 and IL‐10). In the 3D7‐infected systems A–F), when compared to the infected no dose control, 3D7‐infected systems receiving the treatment of 6.0 µg mL^−1^ had a significant increase in IL‐10 on day 7. For the 2.0 µg mL^−1^ treatment groups, a significant decrease in IL‐12 occurred on day 3. The treatment group of 60.0 µg mL^−1^ saw significant increases on day 1 for IL‐6, day 5, and day 7 for IL‐4. Significant decreases were observed for IL‐12 on all days and for TNF‐α, IL‐6, and INF‐γ on day 7. A significant decrease in IL‐10 occurred on day 7. In W2 infected systems G–L), when compared to the infected no dose control, W2‐infected systems receiving the treatment of 6.0 µg mL^−1^, no significant changes were observed. For the 20.0 µg mL^−1^ treatment groups, significant increases occurred for IL‐4 on day 5 and 7, and INF‐γ on day 1. The treatment group of 60.0 µg mL^−1^ saw a significant increase on day 1 for TNF‐α, IL‐6 and INF‐γ, day 3 for IL‐4, day 5 for IL‐4, and day 7 for IL‐4. A significant decrease in IL‐10 occurred on day 7. ^*^ *p* ≤ 0.05, ^**^ *p* ≤ 0.01, ^***^ *p* ≤ 0.001, ^****^ *p* ≤ 0.0001. Data was analyzed via Two‐way ANOVA; Mean ± SEM.

For the 3D7‐infected systems that received chloroquine treatment (**Figure** **6**A–F), a dose‐dependent increase in the regulatory cytokine IL‐4 was observed. This was accompanied by significant dose‐dependent decreases in the inflammatory cytokines TNF‐α, IL‐12, and IL‐6 by day 7. A similar trend for IL‐4 was observed in chloroquine‐treated W2‐infected systems (Figure 6G–L). For the W2‐infected systems, significant increases were observed for TNF‐α, IL‐6, and INF‐γ following chloroquine treatment. This was accompanied by a significant dose‐dependent decrease in IL‐10 by day 7.

**Figure 7:**
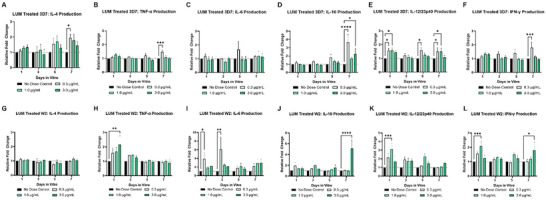
Lumefantrine Effects on Cytokine Production in the Malaria‐on‐a‐Chip Model. Medium collections were performed on days 1, 3, 5, and 7 for monitoring the release of proinflammatory cytokines (TNF‐α, IL‐6, IL‐12, and INF‐γ) and regulatory cytokines (IL‐4 and IL‐10). In 3D7‐infected systems A–F), when compared to the infected no dose control, 3D7‐infected systems receiving the lowest treatment of 0.3 µg mL^−1^ had significant increases in IL‐4, TNF‐α, IL‐6, IL‐10, and INF‐γ, as well as significant increase on days 1 and 5 for IL‐12. For the 1.0 µg mL^−1^ treatment groups, significant increases occurred for IL‐4 on day 7 and IL‐12 and day 1. The highest treatment group of 3.0 µg mL^−1^ saw a slightly significant increase in IL‐10 on day 7. In W2‐infected systems G–L), when compared to the infected no dose control, W2‐infected systems receiving the lowest treatment of 0.3 µg mL^−1^ had significant increases in IL‐6 on days 1 and 3. For the 1.0 µg mL^−1^ treatment groups, significant increases occurred for IL‐12 and INF‐γ on day 1. The highest treatment group of 3.0 µg mL^−1^ saw significant increases in TNF‐α on day 1 and increases in IL‐10 and INF‐γ on day 7. ^*^ *p* ≤ 0.05, ^**^ *p* ≤ 0.01, ^***^ *p* ≤ 0.001, ^****^ *p* ≤ 0.0001. Data was analyzed via Two‐way ANOVA; Mean ± SEM.

For the 3D7‐infected systems that received lumefantrine treatment (**Figure** **7**), when compared to the control group, the inflammatory cytokine IL‐12 was significantly elevated in both the 0.3 and 1.0 µg mL^−1^ doses on day 1. The inflammatory cytokine IL‐12 continued to display significant elevation on day 5 in the low dose, and additional significant increases in the inflammatory cytokines TNF‐α, IL‐12, and IFN‐γ were observed on day 7 in systems treated with the lowest dose of 0.3 µg mL^−1^. In that same group, significant increases of the regulatory cytokines IL‐4 and IL‐10 were observed by day 7. On day 7, IL‐10 was also significantly elevated in the high dose of 3.0 µg mL^−1^. For the W2‐infected systems, as compared to the control group, the lowest dose resulted in a significant increase in IL‐6 on days 1 and 3. The mid dose of 1.0 µg mL^−1^ resulted in significant increases in IL‐12 and IFN‐γ one day after dosing. For the high dose of 3.0 µg mL^−1^, TNF‐ α was significantly elevated on day 1, and IL‐10 and IFN‐γ were significantly higher on day 7 when compared to the control group.

**Figure 8:**
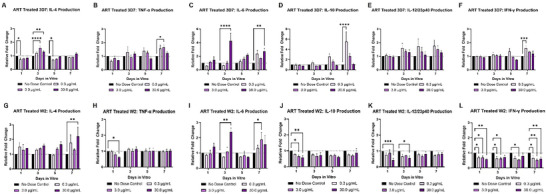
Artesunate Effects on Cytokine Production in the Malaria‐on‐a‐Chip Model. Medium collections were performed on days 1, 3, 5, and 7 for monitoring the release of proinflammatory cytokines (TNF‐α, IL‐6, IL‐12, and INF‐γ) and regulatory cytokines (IL‐4 and IL‐10). In 3D7‐infected systems A–F), when compared to the infected no dose control, 3D7‐infected systems receiving treatment of 0.3 µg mL^−1^ had a significant increase in IL‐4 on day 3, as well as significant increases of IL‐10, IL‐6, and TNF‐α on day 7. For the 3.0 µg mL^−1^ treatment groups, significant increases occurred for IL‐4 on day 3 and TNF‐α on day 7. The treatment group of 30.0 µg mL^−1^ saw significant increases in IL‐4 on day 3, INF‐γ on day 7, as well as IL‐6 on days 3, 5, and 7. A significant decrease in IL‐12 was observed on day 5 for this dose. In W2‐infected systems G–L), When compared to the infected no dose control, W2‐infected systems receiving the lowest treatment of 0.3 µg mL^−1^ had a significant decrease in INF‐γ on days 1, 3, and 7. For the 3.0 µg mL^−1^ treatment groups, significant increases in IL‐6 occurred on day 7, significant decreases occurred for IL‐10 on day 1 and for INF‐γ on days 1, 3, 5, and 7. The highest treatment group of 30.0 µg mL^−1^ saw significant increases in IL‐4 on day 7 and IL‐6 on day 3. Significant decreases occurred in IL‐10 on day 1, IL‐12 on days 1 and 3, and INF‐γ on days 1 and 7. ^*^ *p* ≤ 0.05, ^**^ *p* ≤ 0.01, ^***^ *p* ≤ 0.001, ^****^ *p* ≤ 0.0001. Data was analyzed via Two‐way ANOVA; Mean ± SEM.

For the 3D7‐infected systems that received artesunate treatment (**Figure** **8**A–F), when compared to the control, there was a significant increase in the regulatory cytokine IL‐4 and inflammatory cytokine IL‐6 on day 3 in systems treated with the highest dose of 30.0 µg mL^−1^. IL‐4 was also significantly elevated in the mid‐dose of 3.0 µg mL^−1^. On day 7, there was also a significant increases in regulatory IL‐10 and inflammatory INF‐γ in the 0.3 µg mL^−1^ dose. The mid dose of 3.0 µg mL^−1^ also lent significant increases in TNF‐α production on day 7, and a significant increase in IL‐6 was conferred by the high dose on that day. For the W2‐infected systems treated with artesunate (Figure 8G–L), IL‐10, IL‐12, and INF‐γ were lower than the control group in all doses for all 7 days. IL‐6 was elevated on day 3 and 7 in systems dosed with 30.0 and 3.0 µg mL^−1^, respectively.

### 2.5 Translating Pharmacokinetic and Pharmacodynamic Interactions within the Malaria‐on‐a‐Chip Model to In Vivo Studies

**Figure 9:**
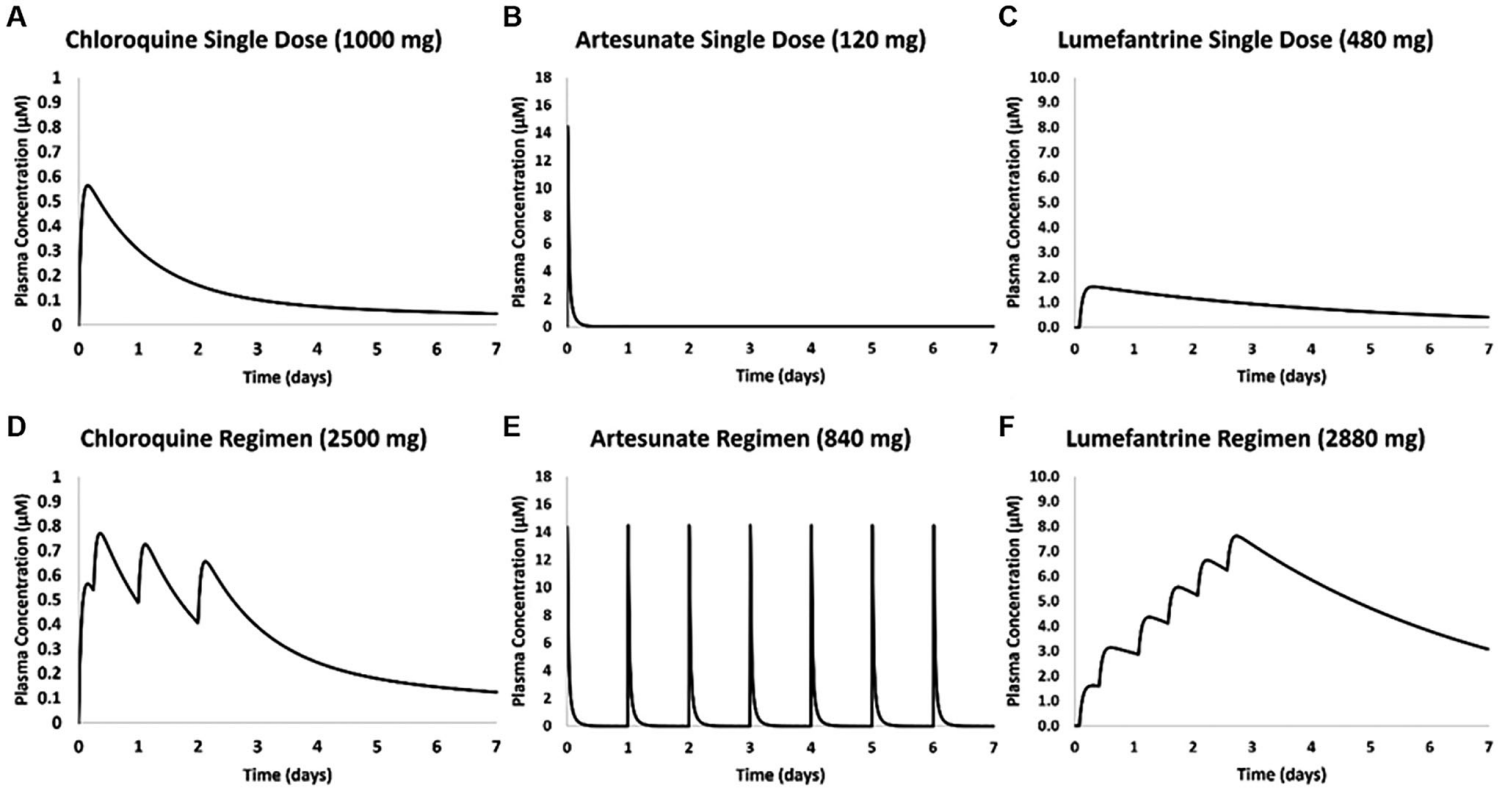
In vivo pharmacokinetic modeling predictions for plasma concentrations of chloroquine, artesunate and DHA, and lumefantrine for single doses and recommended dosing regimens. The single‐dose predictions A–C) were produced by modeling only the first dose of the multi‐dose regimen as suggested by the CDC, to use as a comparison of PK profiles in in vitro to in vivo extrapolation PK/PD modeling. The dosing regimen for chloroquine D) followed the treatment recommendations from the CDC, while the artesunate E) and lumefantrine F) represent the single‐compound components of the recommended combination artesunate‐lumefantrine regimen.

The measured concentrations of each compound within the Malaria‐on‐a‐Chip model and corresponding parasitemia within the system were supplied to the PK/PD model, resulting in drug‐ and strain‐specific PK/PD parameters (**Table** **1**). The parameters for the W2 response to chloroquine resulted in numerically high EC_50_, beyond the range concentration used in the experiment, consistent with the chloroquine‐resistant quality of the W2 strain. The in vivo PK model, developed in Phoenix, generated predictive concentration profiles for chloroquine, artesunate, and lumefantrine, with dosing modeled as both the first dose of a dosing regimen (**Figure** **9**A–C) and the full dosing regimen (Figure 9D–F). While the standard recommended dosing for artesunate and lumefantrine is a combination therapy of the two compounds, artesunate and lumefantrine were modeled separately based on each drug's contribution to the treatment regimen.

**Table 1:** In vitro PK/PD model parameters from the Malaria‐on‐a‐Chip model.

Antimalarial Therapeutic		Chloroquine		Artesunate		Lumefantrine	
*P. falciparum* strain		3D7	W2	3D7	W2	3D7	W2
**Parameters**	k_max_ (hr^−1^)	0.050	0.026	0.026	0.029	0.034	0.039
	EC_50_ (µM)	0.25	3551	0.75	8.6	0.035	0.054
	Hill Coefficient	0.65	0.12	0.11	0.60	0.11	0.08
	Delay (hr^−1^)	32.1	164	1.87	1.70	0.66	0.27

**Figure 10:**
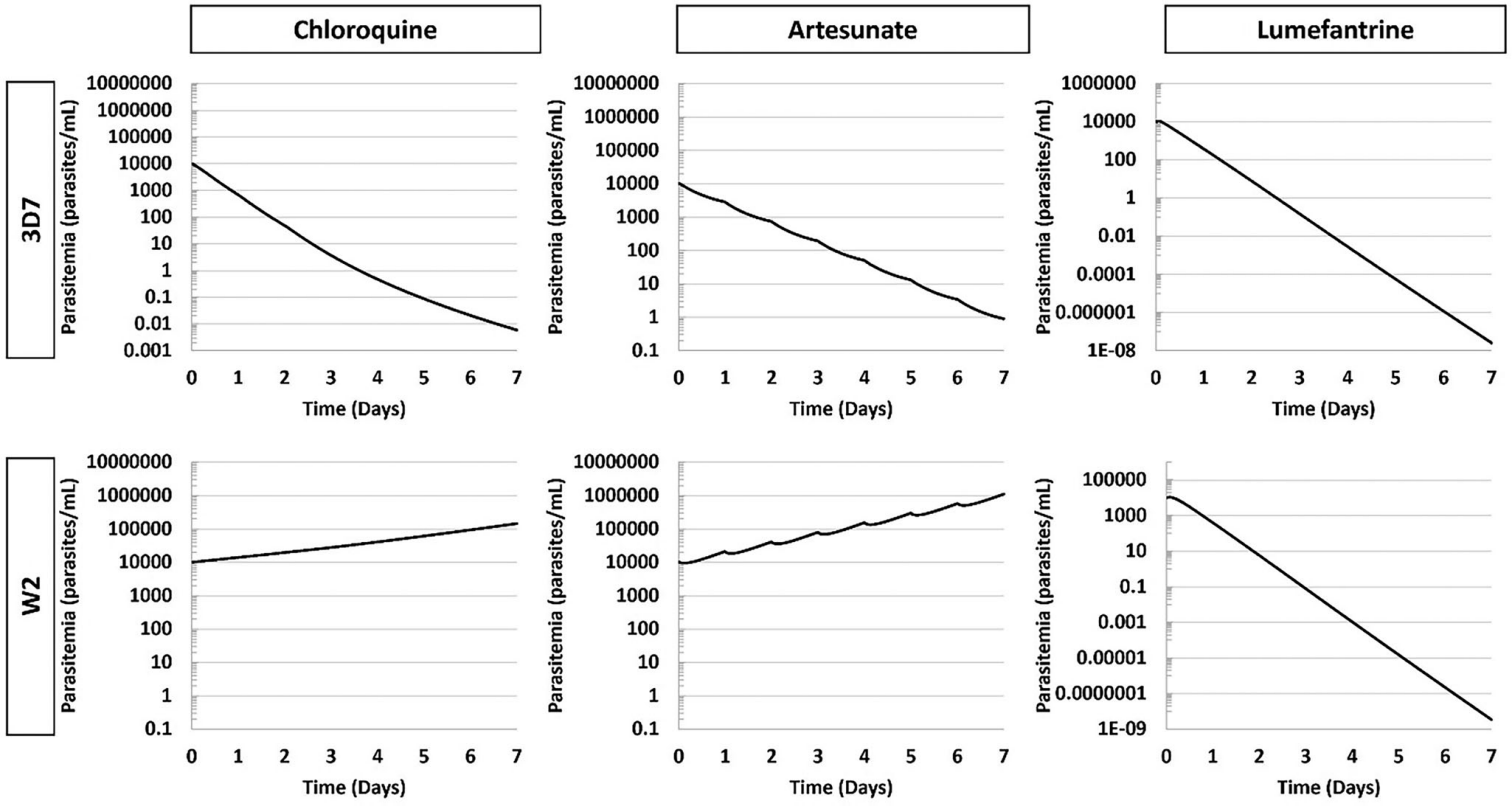
Predicted in vivo parasitemia for the full dosing regimen of antimalarial therapeutics. Predictions for of chloroquine (left), artesunate (middle), or lumefantrine (right) applied to infections of 3D7 (top) and W2 (bottom) P. falciparum strains. An initial value of 10000 parasites mL^−1^ was used for the onset of dosing. The model predicted pronounced strain‐specific effects of chloroquine and artesunate single dosing, with a less pronounced strain‐specific effect of lumefantrine.

When dosed using recommended treatment regimens, or the regime for each component of the combination therapy, the in vitro to in vivo extrapolation PK/PD model predicts prolonged effects of dosing, with strain‐dependent results (**Figure** **10**). In the case of chloroquine treatment of 3D7 infection, the multi‐dose regimen dramatically reduces parasitemia over 7 days, by ≈6 log‐fold. The W2 strain prediction when dosed with chloroquine multi‐dosing regimen was unsurprisingly ineffective, with the parasite number continuing to increase. The artesunate dosing regimen predicted differential effects between 3D7 and W2, with this artesunate regimen predicted to effectively treat only the 3D7 infection. The artesunate was predicted to clear the 3D7 strain in vivo with repeat dosing but only slow the growth of the W2 strain. The model predicted that the lumefantrine regimen would be highly effective for both the 3D7 and W2 strains, resulting in a 12 to 13 log reduction in parasite over 7 days. Because the predicted plasma concentrations for both chloroquine and lumefantrine dosing regimens remain elevated at and presumably beyond 7 days (Figure 10), a continued improvement in parasitemia would be expected.

## 3. Discussion

The work outlined above demonstrates the implementation of a human, multi‐organ, serum‐free, microfluidic platform for the evaluation of antimalarial therapeutic efficacy and off‐target toxicity. This platform recapitulated the disease phenotype by inoculating system medium with infected recirculating erythrocytes, creating a baseline for parasite replication within the system. The resultant spread of infection allowed for observation of pathological outcomes on the organ constructs contained within; this was recognized primarily by the measured detrimental effects on the endothelium as well as the monitoring of immune activity in response to infection.

The previously described Malaria‐on‐a‐Chip model was further implemented for this study.^[^ ^12^ ^]^ This original proof‐of‐concept model was optimized through adjustment of the medium formulation, resulting in a more robust cell viability once integrated into the multi‐organ microphysiological system. In doing so, more refined assessments could be made about organ health in response to infection with *P. falciparum* strains (Figure 1D–F). Additionally, the implementation of flow cytometry introduced higher levels of specificity for parasite quantification and lifecycle monitoring (Figures S6 and S7, Supporting Information). Precise tracking of parasite stages provides an added benefit of identifying effective targets of future antimalarial therapeutics tested in the Malaria‐on‐a‐Chip model.

For the drug treatment employed in this study, the modeling of hyperparasitemia, defined as parasitemia levels reaching 5% or higher, was preferred.^[^ ^13^ ^]^ The highest parasitemia observed for a single 3D7‐infected system was ≈11%. Systems that climbed to these higher rates seemed to reach a threshold parasitemia, with cultures decreasing in parasite levels independent of treatment and before dilution via blood addition occurred. To work within the window of hyperparasitemia and still maintain viable parasites within the model, systems were infected at ≈3% parasitemia on Day 0. An average of ≈6% parasitemia was observed by day 3. Considering the upper limit of 11% in our system, this could potentially explain why the 3D7 culture began to decline by day 4. To maintain the culture, fresh primary erythrocytes were added on day 4, aligning with historical methods of *P. falciparum* culture. After the addition of fresh RBCs, the culture returned to the established growth rate. It is important to note that the resulting parasitemia decreased due to the introduction of these uninfected RBCs. This healthy RBC population skewed the parasite clearance data that was not due to effects of the administered antimalarial therapeutics. To account for this, a dilution factor of 2 was multiplied to the quantified parasitemia for days 5, 6, and 7.

W2‐infected systems were also inoculated with a starting parasitemia of 3%. However, the W2 strain did not replicate as quickly as the 3D7 strain, and a plateau was observed in the latter days of parasite culture. This may be due to culture conditions, with systems maintained in normoxic conditions. In vivo, organs tend to maintain an oxygen concentration that ranges from 3–6%.^[^ ^14^ ^]^ However, most in vitro cell cultures, including the multi‐organ systems in this study, are maintained in incubators with much higher oxygen concentrations of ≈20%. A hypoxic in vitro culture environment is optimal for the asexual replication of *P. falciparum*. Previous in vitro studies have demonstrated that the 3D7 strain can adapt to these changes in atmospheric conditions while the replication rate of the W2 strain begins to decline, explaining the growth trends observed in this study.^[^ ^15^ ^]^

During characterization, the only organ construct with a significant decline in viability attributed to parasitic infection alone were the HUVECs (Figure 1). Recent studies have discussed the detrimental effects of *P. falciparum* on the human vasculature. Exposure to this parasite results in disruption in microcirculation and an increase in shear stress on the endothelium, leading to damage to the glycocalyx.^[^ ^16^ ^]^ This damage to the endothelial glycocalyx allows for increased binding of iRBCs to the endothelial receptors, as well as increased endothelium permeability. This cytoadherence can further disrupt circulation, stimulate local inflammation, and may even result in transmission of parasitic particles to the endothelium, all of which may result in the decline of endothelial cell health.^[^ ^17^, ^18^, ^19^ ^]^ Further validation is necessary to model sequestration, and the observed detrimental effects of *P. falciparum* infection on the endothelium in this model. However, this study indicates the potential this model provides in modeling endothelial injury in response to infection. Continued improvements may provide a more robust model to monitor cytoadherence, which is an application that cannot be obtained in traditional animal models. Additionally, a significant restorative effect in infected endothelium viability was observed following treatment with low concentrations of chloroquine (Figure S8D, Supporting Information). This ameliorative outcome was even more effective with artesunate treatment (Figure S10D, Supporting Information). These results demonstrate that the Malaria‐on‐a‐Chip model can culture healthy endothelium, monitor endothelium that has sustained damage due to infection, and model recovery, provided sufficient treatment administration.

The immune profiling gleaned from this model was an exciting step in understanding the immune response to both infection and treatment in a human‐based preclinical platform. The splenic T and B‐cells contributed to the immune response in this study. While future work is necessary to model the spleen's physical obstacles in this platform, observation of immune activity through cytokine output is currently possible. The overproduction of proinflammatory cytokines TNF‐α, IFN‐γ, IL‐2, IL‐6, and IL‐8 have been associated with the development of cerebral malaria, even when high levels of anti‐inflammatory cytokines such as IL‐10 are expressed.^[^ ^20^ ^]^ Determining splenic cytokine output allowed the ascertainment of spleen function and effects of antimalarial treatment on cytokine production.

Interestingly, W2‐infected systems treated with artesunate resulted in significant decreases in cytokine release profiles when compared to untreated systems. As a known immunosuppressant,^[^ ^21^ ^]^ artesunate restricts the activation and proliferation of lymphocytes^[^ ^22^ ^]^ and downregulates production of pro‐inflammatory cytokines.^[^ ^23^ ^]^ The combination of W2 infection with artesunate treatment may have contributed to the dose‐dependent decline in spleen viability. This synergistic effect on viability and function further supports previous literature findings suggesting off‐target effects in the spleen.

Comparisons to in vivo administration of the drug is a slight limitation of this study. Considering artesunate's half‐life of ≈45 min, effects are short‐lived, so it is often co‐administered with an oral medication such as amodiaquine or mefloquine to prevent recrudescence and development of drug resistance.^[^ ^24^ ^]^ A combination treatment regimen typically lasts 3 days so that artesunate's effects persist through two asexual cycles of the parasite. This ensures that the partner drug must only clear the small remaining portion of the parasite burden. Dosage is weight‐dependent, with recommendations between 2–10 mg kg^−1^ daily for artesunate.^[^ ^25^ ^]^ For lumefantrine, based on in vitro observations and clinical trials, a 1:6 ratio (20 mg artemether and 120 mg lumefantrine) is the ideal concentration due to its high effectiveness and lack of significant toxicity in clinical trials. This combination is typically administered orally in a fixed combination tablet form with artemether 80 mg and lumefantrine 480 mg. Four doses are given consecutively at hours 0, 8, 24, and 48, for a total of 320 mg artemether and 1920 mg lumefantrine.^[^ ^26^, ^27^ ^]^ Furthermore, results from HPLC‐LCMS quantification revealed a limitation in the ability to study lumefantrine within the Malaria‐on‐a‐Chip model. High absorption into the PDMS, coupled with low solubility in DMSO (1.625 mg mL^−1^), meant that there would not be a chance to deliver the recommended therapeutic dose of lumefantrine within the system.

Given the outlined in vivo dosing regimen, this model administered treatment as monotherapy in a single bolus dose. As this model continues development, it will be necessary to observe parasite recrudescence following treatment, to track both treatment effectiveness and potential resistance to existing and novel therapies. Delivering a single dose allowed for monitoring of the parasite's ability to return following treatment, demonstrating that this platform can be an effective model for detecting recrudescence utilizing population dynamics.

Translational mechanistic models for malaria seek to utilize data obtained in vitro to produce effective treatments for patients with malaria, with particular emphasis on populations with malnutrition and concomitant viral infections. The combination of varying levels of nutrition and age, particularly among children, creates a particular difficulty for determining the pharmacokinetics of administered treatments within those patients. PK/PD translational modeling to develop digital twins, incorporating physiologically based pharmacokinetics (PBPK), is uniquely suited to address this challenge, as the models are developed to assess a range of patient conditions, instead of relying on limited clinical studies. We have shown the ability to and utility of generating PK/PD relationships within the Malaria‐on‐a‐chip system, by creating a digital twin of each system, and coupling with PBPK models to predict clearance of parasite in humans. The Malaria‐on‐a‐chip system can be modified with disease state tissues, including impaired liver that would affect the pharmacokinetics of clearance and production of active metabolites, or with concomitant infections, to investigate the PK/PD relationships for these malnourished or co‐infected populations for both efficacy and off‐target toxicity. The Malaria‐on‐a‐chip can be further utilized to test drug–drug combinations for these populations with nearly unlimited flexibility. Applying these PK/PD relationships alongside a PBPK approach that models these difficult patient populations can further define patient drug PK relationships to produce digital twins for patient groups or even individual patients for improved treatment. Refinement of the PBPK models is critical for predicting patient pharmacokinetics, however, the translation of the pharmacokinetics to patient outcomes relies on the PK/PD modeling of the experimentally generated data.

Currently both PBPK and PK/PD models require clinical data for each compound, including measuring patient plasma levels of compounds and determination of parasitemia. Machine learning‐based models based on structure‐property relationships show great promise for predicting the pharmacokinetic behavior in vivo without clinical PK data, but physiological responses for both efficacy and toxicity are more complex, not only due to unknown or undetermined mechanistic effects but also due to the complex time‐dependence of these responses. The PK profiles of chloroquine, artesunate, and lumefantrine in vivo were based on existing PK models for those compounds derived from human patient data. The efficient prediction of the PK of untested compounds is an entire area of study, which is making dramatic improvements utilizing machine learning and physiologically based pharmacokinetic modeling (PBPK), with efforts existing to provide a toolbox from current malaria compounds to aid modeling tools for in vitro to in vivo translation using digital medical twins. This work forms the basis for future exploration utilizing the Malaria‐on‐a‐chip device to refine and confirm predictions from machine learning‐based models for predicting compound characteristics, for creation of more detailed digital medical twins, and evaluation of treatment regimens for other populations, such as children and elderly, especially those with malnourishment or other viral infections more common in populations at risk for malaria.

## 3 Experimental Section

### System Fabrication and Assembly

The Malaria‐on‐a‐Chip model contains three separate housing chambers for the different organ constructs within the system: liver, spleen, and endothelium. The chambers were all interconnected allowing for the circulation of blood, drugs, and medium throughout the system. The housing pieces were 6 mm‐thick clear cast acrylic sheets separated by four 0.5 mm‐thick polydimethylsiloxane (PDMS) elastomer sheet layers, both of which were cut using a Boss Laser LS1420 65 W CO_2_ laser cutter. The precise laser cutting of the housings and gaskets created specific locations for the coverslips to be placed along with the designation of the microfluidic pathway of the system. Prior to system assembly, the individual housing and gasket pieces were cleaned via sonication, disinfected with 70% IPA, and passivated using a 3% Bovine Serum Albumin (BSA) in 1x PBS solution. On the day of assembly, the passivation solution was removed immediately before placing the three coverslips (one for each cell type) into their respective chamber on the bottom housing unit. This step was immediately followed by the addition of multi‐organ serum‐free medium (MOSM) on top of the coverslips. After the addition of medium, the top housing unit was aligned carefully, and the two units were screwed together using a torque screwdriver set to 40‐in‐oz. Once assembled, the systems were incubated on a rocking platform set at a defined angle to ensure the recirculation of medium and blood throughout the systems for the duration of the studies.

### Primary Human Hepatocyte Culture

Hepatocytes were obtained from Novabiosis (Lot #BEI) and thawed and cultured followed manufacturer protocols. Cells were plated 6 days prior to system assembly. Pre‐plating, coverslips were coated with a collagen solution (rat tail collagen I [Sigma, Cat no. C5533], dPBS [Corning, Cat#21‐031‐CV], Acetic Acid [Sigma–Aldrich, cat no. A6283‐1L]). Cells were maintained using Novabiosis Maintenance Medium (Novabiosis, cat no. 7111) according to the manufacturer protocol. 24 h prior to system assembly, coverslips were transferred to new plates and a collagen topcoat solution was applied to each coverslip. Each coverslip was examined via phase microscopy prior to assembly for healthy morphology and confluence.

### Human Umbilical Vein Endothelial Cell Culture

Human Umbilical Vein Endothelial Cells (HUVECs) were obtained from Lonza (Lot#0000636514) and cultured following manufacturer protocols. These cells were thawed and plated four days prior to system assembly onto rat tail collagen (Millipore, Cat. no. 08–115) coated coverslips. Vendor specific medium, EGM‐2 (Lonza, Cat. no CC‐3156) supplemented with growth factors (Lonza, Cat. no CC‐4176) was used for the maintenance of these cells.

### Primary Human Splenocyte Culture

Human splenocytes (BIOIVT, Cat no. 0107871) were thawed and seeded one day prior to system assembly. Cells were cultured using splenocyte base medium, composed of RPMI‐1640 (Thermofisher, Cat no. 2340062) supplemented with heat‐inactivated FBS (Sigma, Cat no. F4135), pen/strep (Sigma, Cat no. P433), and β‐Mercaptoethanol (Sigma, Cat no. M6250). A Hydrogel solution was used for plating, composed of rat tail collagen I (Sigma, Cat no. C5533) and MEM 10X (Thermofisher, Cat no. 11430030). The pH of the solution was adjusted using 1N NaOH prior to the addition of cell suspension. Cells were plated on coverslips, and 2 h post‐plating a full medium change was performed with stimulation medium containing human recombinant GM‐CSF (Stem Cell, Cat no. 78190), human recombinant IL‐4 (Stem Cell, Cat no. 78045.1) and human recombinant IL‐2 (Stem Cell, Cat no. 78036.1).

### Plasmodium Falciparum Culture

Following the original method for continuous culture of *Plasmodium falciparum*,^[^ ^28^ ^]^ the cells were cultivated in RPMI 1640 medium (Thermofisher, cat no. 2340062) supplemented with 25 mM HEPES buffer (Thermofisher Scientific, cat no. 15‐630‐080). 5% Albumax II (Thermofisher, cat no. 11020021) and hypoxanthine (Sigma–Aldrich, cat no. H9377‐5G) were also added to the medium as a serum replacement.^[^ ^29^ ^]^ The medium was buffered using NaHCO3; the pH was assessed every other day and adjusted as necessary to achieve a pH between 7.2–7.45. Medium changes were performed daily by centrifuging cultures (400 x g, 5 min, 22 °C) and replacing supernatant with fresh medium. While some studies have shown 3D7 can maintain growth in both hyperoxic (5% CO_2_, 21% O_2_, 74% N_2_) and hypoxic environments, the growth of other strains can stall in hyperoxic environments.^[^ ^15^ ^]^ With both strains (3D7, W2), all cultures were kept in a tri‐gas incubator where gas balance was maintained at 5% O_2_, 5% CO_2_, and 90% N_2_ at 37 °C. Time outside of the incubator was minimized, and each flask was gassed for 5 min after medium change. Sub‐cultures were performed every 2 weeks to lower parasitemia to 3%. To facilitate continued parasite replication, fresh red blood cells were added to the medium at 3% every four days.

### Multiorgan Culture in Malaria‐on‐a‐Chip Platform

Assembly and dosing of the systems (day 0) was completed using MOSM‐2 containing stimulation factors IL‐2 (STEMCELL Technologies, cat. no. 78036.1), IL‐4 (STEMCELL Technologies, cat. no. 78045.1), and GM‐CSF (STEMCELL Technologies, cat. no. 78190). A 30% medium change was performed every day after assembly, and the medium contained stimulation factors for the two days following assembly. Medium collections were performed the day following assembly and every other day thereafter. These samples were stored at −80 °C until analysis. Freshly washed and isolated healthy red blood cells were added to the systems on day 4.

### Blood Addition

As previously described, iRBCs were administered to the microfluidic system for a total hematocrit of 3%. To maintain the parasite culture in the Malaria‐on‐a‐Chip platform, an additional volume of freshly isolated RBCs were added to the system on day 4, adherent to traditional in vitro culture protocols.

### CYP3A4 Assay

Following system disassembly, hepatocyte coverslips were placed in a 24‐well plate and washed with PBS prior to addition of the Luc‐IPA reagent. Using the P450‐Glo CYP3A4 Assay with Luciferin IPA (Promega, cat no. V9002), Luc‐IPA was diluted with Dulbecco's Modified Eagle Medium (Thermofisher, cat no. A1443001), and this solution was added to each washed coverslip. A blank well was also prepared. The plate was incubated at 37 °C for 1 h, then the full volume of reagent was collected into sample tubes. For the second reaction, 50 µL of each collected sample was added to a 96‐well plate. The Luciferin Detection Reagent (Promega, cat no. V9002) was reconstituted with the provided Reconstitution Buffer. Then, 50 µL of this reagent was added to each sample well. The plate was incubated at room‐temperature for 20 min. The luminescence was measured using a plate reader.

### MTT Assay

MTT absorbance was measured immediately following the CYP assay. MTT reagent (Invitrogen, cat no. M6494) diluted in hepatocyte maintenance medium was delivered to each sample well. The plate was incubated at 37 °C for 1 h before the solution was removed and discarded. A solubilization solution was prepared using dimethyl sulfoxide, 10% SDS, and acetic acid. The solubilization solution was added to each well, and the plate was placed on a plate shaker for 5 min at 580 RPM. This step was repeated until the formazan crystals were fully dissolved in the solution. Each sample was collected and dispensed into a 96‐well plate. The concentration of formazan was determined by optical density using a plate reader at 540 and 720 nm.

### alamarBlue Assay

An alamarBlue assay was performed on the splenocyte and HUVEC cultures. Coverslips were transferred to 12‐well plates and washed three times with PBS. A 10% alamarBlue (Thermo Fisher, cat no. DAL1100) solution in system culture medium was added to each well. The plates were incubated at 37 °C for 24 h. After this incubation, samples were added in triplicate to a 96‐well plate. The plate was read using a microplate reader at excitation fluorescence of 560 nm and emission fluorescence of 590 nm.

### Multiplex ELISA

A custom‐ordered ProcartaPlex Luminex ELISA kit (lot #347833‐000) from Thermofisher and the Magpix Luminex system (SN MAGPX16176702) were used to determine concentrations of the cytokines TNF‐α, IFN‐γ, IL‐6, IL‐12, IL‐4 & IL‐10. Cell culture supernatants from each system were collected on days 1, 3, 5 and 7 of experimentation and stored at −80 °C until analysis. The viability of each spleen component was analyzed via alamarBlue assay. The sample from each dose's most viable spleen was used for cytokine analysis. Samples were processed following manufacturer protocols. In brief, samples were thawed on ice, clarified via centrifugation, and added to the provided 96 well plate in duplicate. Washes were performed by hand using the provided wash buffer and a multichannel pipette. 1:4 dilution standards were prepared using the provided stock vials, with an additional bottom dilution prepared to lower standard detection limits. Data was analyzed using the companion Procartaplex analysis application provided by Thermofisher.

### Drug Preparation and Addition to Systems

Drug doses were prepared on the day of system assembly. Powder chloroquine phosphate (Sigma, Cat no. PHR1258), artesunate (Sigma, Cat no. 1042850) and lumefantrine (Sigma, Cat no. 1370746) were weighed out using an analytical balance, and the compounds were reconstituted in the appropriate solvent. Chloroquine was reconstituted in culture medium while lumefantrine and artesunate were reconstituted in DMSO, for final stock concentrations of 10.0, 1.625, and 14.0 mg mL^−1^ respectively. Effective doses were derived from the Malaria in the United States: Treatment Tables from the CDC for each compound. Dose concentrations were then converted from mg kg^−1^ of body weight to µg/mL^−1^ to model the dose within the microfluidic platform. Doses were prepared for each compound using the reconstituted stock solutions. Chloroquine doses were prepared to deliver doses of 0.2, 6.0, 20.0, 60.0, 200.0, 600.0, and 2000.0 µg mL^−1^. Lumefantrine doses were prepared to deliver doses of 0.3, 1.0, and 3.0 µg mL^−1^. Artesunate doses were prepared to deliver doses of 0.03, 0.3, 3.0, 30.0, 90.0, and 150.0 µg mL^−1^. Each dose was concentrated to be delivered in a single bolus dose to the system volume. The doses were delivered to the systems through a medium change, removing a volume of culture medium from the system prior to adding the dose.

### High Pressure Liquid Chromatography (HPLC) and Mass Spectrometry (MS)

The analysis was performed using two instruments for the analysis of lumefantrine (LUM) and chloroquine (CHQ), while artesunate (ART) was analyzed using one instrument. The first system consists of a Waters Acquity Ultra‐Performance Liquid Chromatography Xevo Triple Quadrupole Mass Spectrometer. The second system consists of a Thermo Scientific Vanquish Ultra‐High‐Performance Liquid Chromatography TSQ Altis Plus Triple Quadrupole Mass Spectrometer. For the analysis of LUM and CHQ, the mobile phase consisted of Methanol 0.1% Formic Acid and Water 0.1% Formic Acid with a flow rate of 0.5 mL min^−1^ on the Water System and a flow rate of 0.3 mL min^−1^ on the Thermo System. For the analysis of ART, the mobile phase consisted of Methanol 0.1% Formic Acid and 10 nm Ammonium Formate with a flow rate of 0.2 mL min^−1^. Samples were injected onto the column using an injection volume of 5 µL. Only UPLC‐grade solvents and reagents were used in this analysis.

The source of ionization for all analytes was electrospray ionization (ESI) in positive mode, and the analysis was performed using multiple reaction monitoring (MRM) transitions. Calibration curves were constructed using a range of calibration standards for each analyte, between 0.01 and 5 µm using a 1/x^2^‐weighting calibration. Non‐match matrix calibration standards were prepared in Methanol mixed with equal amounts of internal standard mix. 0.3 µm of internal standard was added into each vial. The internal standard mix consisted of 80% Water, 20% Methanol, and 0.1% Formic Acid. The matrix effect was calculated by comparing the response of the analytes in the presence of the matrix to that in the absence of the matrix as shown in Equation 1.

(1) $$\begin{array}{cc} & \mathrm{Matr}\mathrm{ix}\;\mathrm{Effe}\mathrm{ct}\left(\%\right)\\ & \;=\left[\frac{\begin{array}{c} \left(\mathrm{Slope}\;\mathrm{anal}\mathrm{ytic}\mathrm{al}\;\mathrm{curve}\;stan\mathrm{dard}\;\mathrm{in}\;\mathrm{matr}\mathrm{ix}\right)\\ -\left(\mathrm{Slope}\;\mathrm{anal}\mathrm{ytic}\mathrm{al}\;stan\mathrm{dard}\;\mathrm{in}\;\mathrm{solv}\mathrm{ent}\right) \end{array}}{\mathrm{Slope}\;\mathrm{anal}\mathrm{ytic}\mathrm{al}\;\mathrm{curve}\;stan\mathrm{dard}\;\mathrm{in}\;\mathrm{solv}\mathrm{ent}}\right]\times 100 \end{array}$$

The match‐matrix standards and samples prepared were prepared in MOSM‐2 medium used during the experiments and were placed into a centrifuge tube to undergo a protein precipitation procedure by placing the samples in the benchtop centrifuge and spin down using the highest speed setting for 5 min at 4 °C. The protein precipitation step was performed to remove proteins, endogenous components, and other potential interferents from the samples. The matrix effect was attributed to the protein precipitation procedure used for sample preparation; however, the specific analyte responsible for ion suppression was not determined. Lidocaine was used as the internal standard for LUM and CHQ while Diclofenac was used as the internal standard for ART. The final concentration of match‐matrix standards aligned with the non‐match matrix calibration standards. Data acquisition, processing, and quantitation were performed using Masslynx in Waters and Thermo software. Method development and optimization were performed for these analytes. The quantitation limit for each analyte was noted at 0.01 µm for each analyte. A quality control standard at the midpoint of 1.0 µm was analyzed every 15–20 injections with an acceptance criterion of 70–130%.

### Parasite Quantification – Giemsa Stain

A blood smear was performed for each system daily. The smear was allowed to air dry and fixed in methanol. The fixed slide was placed in a dilute Giemsa Stain (Fisher Scientific, cat no. 23–264983) for 45 min and then rinsed with DI water and allowed to air dry in a vertical position. Slides were stored at 4 °C until imaging via phase microscopy. Three sections of each slide were imaged, with a minimum of 300 cells counted per image. The number of infected cells was divided by the total number of cells to determine the parasitemia percentage of each imaged section. The overall parasitemia of each sample was determined by averaging the percentages calculated for each of the three sections imaged.

### Parasite Quantification – Flow Cytometry

From the culture flask or system of interest, medium was collected and pipetted into a microcentrifuge tube containing RPMI 1640 medium (Thermofisher, Cat#2340062). This solution was homogenized by pipetting up and down, and a manual cell count was performed via hemocytometer to ensure a density ≥ 1.0 × 10^6^ cells µL^−1^ solution. This solution was then added in quadruplicate to wells of a v‐bottom 96 well plate (Millipore, Cat#M8185), and cells were pelleted by centrifugation (400 x g, 5 min, 22 °C). The supernatant was removed by quickly inverting the plate, and cells were washed once with dPBS via centrifugation. After removing supernatant, one well of each sample was resuspended with dPBS (Corning, Cat#21‐031‐CV), and three wells of each sample were stained by resuspending with dilute SYBR Green I (Invitrogen, Cat no. S7563). The plate was incubated at 37 °C for 20 min, centrifuged, and the supernatant was removed. Cells were washed twice more by centrifugation with PBS. Cell pellets were then resuspended in dPBS, and the plate was immediately run on the flow cytometer. Stopping rules were applied such that 300 000 events would be recorded per sample well. In the acquisition settings the gain for FSC, SSC, and FITC was 120. Samples were gated on a dot plot of FSC‐A v FSC‐H to identify the RBC population by size for doublet exclusion. That gated population (P1), was then plotted on a FIT‐C histogram. A line segment was applied to record all P1 measurements from 10^4^ to 10^6^ to identify the iRBC population (P2). The percentage of P2 for the uninfected RBC samples was subtracted from the iRBC samples as a blank to exclude any fluorescence of the uninfected RBC population. The average of each well was then obtained to determine the parasitemia for the collection timepoint.

### Pharmacokinetic (PK)‐Pharmacodynamic (PD) Modeling

PK/PD modeling for extrapolating the malaria‐on‐a‐chip data for human predictions followed a process of determining PK/PD relationships in the in vitro system, modeling the PK in vivo, and applying the in vitro PK/PD relationships with the in vivo PD models (Figure S5, Supporting Information). The in vitro PK models were developed by modeling a bi‐exponential function including convective mixing and drug material adsorption to experimental drug concentration data in the malaria‐on‐a‐chip system quantified via HPLC‐MS. In vitro population PK/PD relationships for the three drugs were developed using a modified sigmoidal E_max_ model for anti‐infectives against bacterial strains.^[^ ^30^ ^]^ In vivo pharmacokinetics were adapted from clinical patient literature utilizing compartmental models for chloroquine,^[^ ^31^ ^]^ artesunate,^[^ ^32^ ^]^ and lumefantrine.^[^ ^33^ ^]^ In vivo PD predictions were generated utilizing the scaled in vitro rate constants and the in vivo PK profiles generated for current recommended dosing protocols. The digital twins are represented in population dynamics models. The Supporting information contains additional details on the model, software, algorithms, and statistical validation of these models.

### Statistical Methods

Values are expressed as the mean standard error of the mean (SEM) of a minimum of 3 independent experimental replicates. Data points were collected such that significant differences were able to be observed in the data; *n* = 9 – 12 depending on the condition being presented. Two‐way ANOVA was employed for the analysis of parasitemia when analyzing approaches to parasite quantification (Giemsa v Flow cytometry), as well as when analyzing effects of antimalarial therapeutics on parasite concentration in the model. One‐way ANOVA was implemented when analyzing the viability of the organ constructs in the system with value comparisons drawn between treatment groups to the no dose control. Asterisks were used to indicate the ranges of p values (^*^ *p* ≤ 0.05, ^**^ *p* ≤ 0.01, ^***^ *p* ≤ 0.001, ^****^ *p* ≤ 0.0001).

## Conflict of interest

James J. Hickman has ownership interest and is Chief Scientist and member of the Board of Directors in a company that may benefit financially as a result of the outcomes of the research or work reported in this publication. All other authors declare no financial interests.

## Supporting information



Supporting Information

## Data Availability

The data that support the findings of this study are available from the corresponding author upon reasonable request.;

## References

[1] C. A. Moxon , M. P. Gibbins , D. McGuinness , D. A. Milner , Jr. , M. Marti , Annu. Rev. Pathol. 2020, 15, 315. 31648610 10.1146/annurev-pathmechdis-012419-032640

[2] C. V. Plowe , Malar. J. 2022, 21, 104. 35331231 10.1186/s12936-022-04115-8 PMC8943514

[3] K. Venugopal , F. Hentzschel , G. Valkiunas , M. Marti , Nat. Rev. Microbiol. 2020, 18, 177. 31919479 10.1038/s41579-019-0306-2 PMC7223625

[4] L. H. Miller , D. I. Baruch , K. Marsh , O. K. Doumbo , Nature 2002, 415, 673. 11832955 10.1038/415673a

[5] P. H. David , M. Hommel , L. H. Miller , I. J. Udeinya , L. D. Oligino , Proc. Natl. Acad. Sci. 1983, 80, 5075. 6348780 10.1073/pnas.80.16.5075 PMC384191

[6] V. Bronte , M. J. Pittet , Immunity 2013, 39, 806. 24238338 10.1016/j.immuni.2013.10.010 PMC3912742

[7] R. Suwanarusk , B. M. Cooke , A. M. Dondorp , K. Silamut , J. Sattabongkot , N. J. White , R. Udomsangpetch , J. Infect. Dis. 2004, 189, 190. 14722882 10.1086/380468

[8] D. Ghosh , J. S. Stumhofer , J. Leukoc. Biol. 2021, 110, 753. 33464668 10.1002/JLB.4RI1020-713R PMC8518401

[9] M. M. Mota , A. Rodriguez , BioEssays 2002, 24, 149. 11835279 10.1002/bies.10050

[10] P. K. Rathod , T. McErlean , P. C. Lee , Proc. Natl. Acad. Sci. 1997, 94, 9389. 9256492 10.1073/pnas.94.17.9389 PMC23200

[11] E. Hughes , E. Wallender , A. Mohamed Ali , P. Jagannathan , R. M. Savic , Clin. Pharmacol. Ther. 2021, 110, 926. 33763871 10.1002/cpt.2238 PMC8518425

[12] M. J. Rupar , T. Sasserath , E. Smith , B. Comiter , N. Sriram , C. J. Long , C. W. McAleer , J. J. Hickman , Sci. Rep. 2023, 13, 10509. 37380653 10.1038/s41598-023-35694-4 PMC10307889

[13] A. Trampuz , M. Jereb , I. Msuzlovic , R. M. Prabhu , Crit. Care 2003, 7, 315. 12930555 10.1186/cc2183 PMC270697

[14] J. Zhang , H. Tavakoli , L. Ma , X. Li , L. Han , X. Li , Adv. Drug Deliv. Rev. 2022, 187, 114365. 35667465 10.1016/j.addr.2022.114365

[15] S. Duffy , V. M. Avery , Int. J. Parasitol. Drugs Drug Resist. 2017, 7, 295. 28738214 10.1016/j.ijpddr.2017.07.001 PMC5522918

[16] U. Schott , C. Solomon , D. Fries , P. Bentzer , Scand. J. Trauma Resusc. Emerg. Med. 2016, 24, 48. 27068016 10.1186/s13049-016-0239-y PMC4828893

[17] V. Introini , A. Carciati , G. Tomaiuolo , P. Cicuta , S. Guido , J. R. Soc. Interface 2018, 15, 20180773. 30958233 10.1098/rsif.2018.0773 PMC6303788

[18] C. Hempel , E. M. Pasini , J. A. L. Kurtzhals , Trends Mol. Med. 2016, 22, 453. 27161599 10.1016/j.molmed.2016.04.004

[19] C. Utter , A. E. Serrano , J. W. Glod , M. J. Leibowitz , Yale. J. Biol. Med. 2017, 90, 183. 28656007 PMC5482297

[20] W. L. Mandala , C. L. Msefula , E. N. Gondwe , M. T. Drayson , M. E. Molyneux , C. A. MacLennan , Clin. Vaccine Immunol. 2017, 24, 00533. 10.1128/CVI.00533-16 PMC5382826 28122790

[21] L. Lin , Z. Tang , Z. Shi , Q. Guo , H. Xiong , J. Immunol. Res. 2022, 2022, 9591544. 35178460 10.1155/2022/9591544 PMC8844150

[22] P. Veerasubramanian , P. Gosi , C. Limsomwong , D. S. Walsh , Southeast Asian J. Trop. Med. Public Health 2006, 37, 838. 17333724

[23] Z. Li , X. Shi , J. Liu , F. Shao , G. Huang , Z. Zhou , P. Zheng , FASEB J. 2019, 33, 8241. 30916998 10.1096/fj.201900146R

[24] K. M. Hess , J. A. Goad , P. M. Arguin , Ann. Pharmacother. 2010, 44, 1250. 20551300 10.1345/aph.1M732

[25] Organization WH ., Guidelines for the Treatment of Malaria, 3rd ed, World Health Organization, Geneva 2015.

[26] N. J. White , M. van Vugt , F. Ezzet , Clin. Pharmacokinet. 1999, 37, 105. 10496300 10.2165/00003088-199937020-00002

[27] F. Ezzet , M. van Vugt , F. Nosten , S. Looareesuwan , N. J. White , Antimicrob. Agents Chemother. 2000, 44, 697. 10681341 10.1128/aac.44.3.697-704.2000 PMC89749

[28] W. Trager , J. B. Jensen , Science 1976, 193, 673. 781840 10.1126/science.781840

[29] S. L. Cranmer , C. Magowan , J. Liang , R. L. Coppel , B. M. Cooke , Trans. R. Soc. Trop. Med. Hyg. 1997, 91, 363. 9231219 10.1016/s0035-9203(97)90110-3

[30] W. Treyaprasert , S. Schmidt , K. H. Rand , U. Suvanakoot , H. Derendorf , Int. J. Antimicrob. Agents 2007, 29, 263. 17194570 10.1016/j.ijantimicag.2006.08.049

[31] A. N. Abd‐Rahman , L. Marquart , N. Gobeau , A. Kümmel , J. A. Simpson , S. Chalon , J. J. Möhrle , J. S. McCarthy , Clin. Pharmacol. Ther. 2020, 108, 1055. 32415986 10.1002/cpt.1893 PMC7276750

[32] Q. Li , L. R. Cantilena , K. J. Leary , G. A. Saviolakis , R. S. Miller , V. Melendez , P. J. Weina , Am. J. Trop. Med. Hyg. 2009, 81, 615 19815876 10.4269/ajtmh.2009.09-0150

[33] S. F. Hietala , A. Martensson , B. Ngasala , S. Dahlström , N. Lindegårdh , A. Annerberg , Z. Premji , A. Farnert , P. Gil , A. Björkman , M. Ashton , Antimicrob. Agents Chemother. 2010, 54, 4780. 20713675 10.1128/AAC.00252-10 PMC2976134

[34] Centers for Disease Control and Prevention (U.S.) . Malaria in the United States: Treatment Tables. 2023, https://stacks.cdc.gov/view/cdc/131219.

[35] N. J. White , Malar. J. 2017, 16, 88. 28231817 10.1186/s12936-017-1731-1 PMC5324257

