# Supplementary material for: Translation of a Human‐Based Malaria‐on‐a‐Chip Phenotypic Disease Model for In Vivo Applications
Source: Adv Sci (Weinh). 2025 Jul 21;12(38):e05206. doi: 10.1002/advs.202505206 (PMC12520463; doi:10.1002/advs.202505206)
Supplement: Supplementary file 1 — Supporting Information [file ADVS-12-e05206-s001.docx]

Supporting Information

**Translation of a Human-Based Malaria-on-a-Chip Phenotypic Disease Model for In Vivo Applications**

*Michael J. Rupar^1^, Hannah M. Hanson^1^, Brianna L. Botlick^1^, Narasimhan Sriram^1^, Stephanie Rogers^1^, Justin Zuniga^2^, Zhanhe Liu^1^, Steven J. Trimmer^1^, Joseph M. Ciurca^1^, Christopher J. Long^1^, Christopher W. McAleer^1^, Stephan Schmidt^3^, Paola Favuzza^4^, Philip Lowe^4^, Nathalie Gobeau^4^, James J. Hickman^1^**

**Malaria-on-a-chip model design**

The schematic of the Malaria-on-a-Chip model (**Figure S1**) below depicts the model design which allows for 3 separate organ chips to be contained within a continuous fluidic pathway. Top and bottom housing components were fabricated with top and bottom acrylic pieces that sandwich together PDMS gaskets which define the organ chip compartments and fluid pathway.


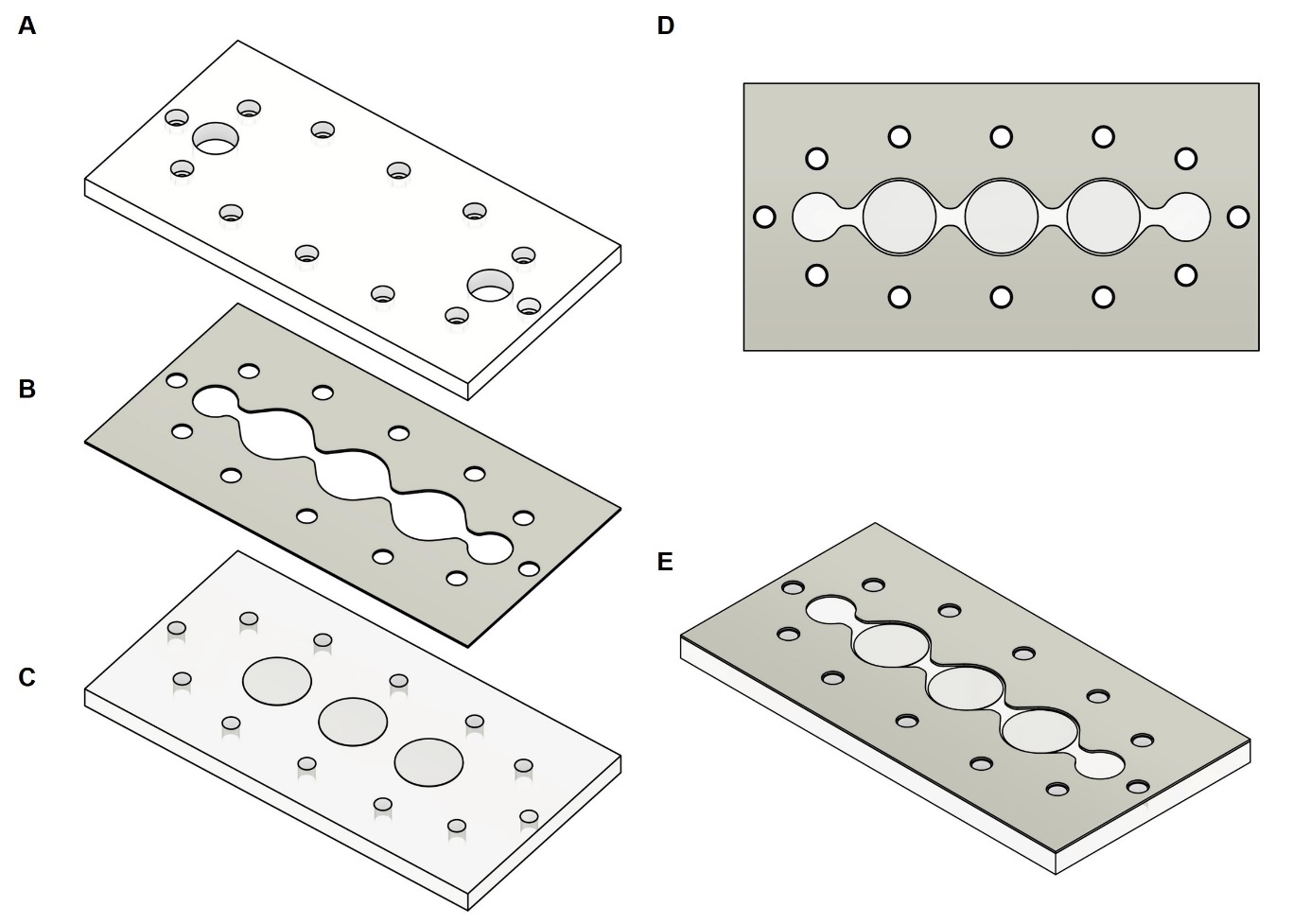


**Figure S1. Malaria-on-a-Chip Schematics.** The Malaria-on-a-Chip system design consisted of an (A) acrylic top, (B) PDMS gaskets, and (C) an acrylic bottom. Coverslips were laid on the bottom housing, medium added atop the coverslips, and 5/16” screws were then used to assemble the housing components together with the PDMS gaskets sandwiched between the top and bottom pieces. (D) Birds eye view of the Malaria-on-a-Chip system and (E) an oblique view.

**Maximum Tolerable Dose (MTD) and No Observable Adverse Effects Level (NOAEL)**

Following the experimental studies to determine the free drug in the system, studies were implemented to determine the max tolerable dose (MTD) and no observable adverse effects level (NOAEL) for each of the compounds used in the Malaria-on-a-Chip model. These systems consisted of one of two options: the first being a multi-organ system containing hepatocytes, splenocytes, and HUVECs; the second contained only red blood cells. These were separated so that the morphology of the functional cells could be observed using phase imaging.

For chloroquine-treated systems (**Figure S2**), phase imaging showed dramatic decline in cell viability for both the hepatocytes and HUVECs from day 1 to day 7 in a dose dependent manner. Toxicity levels became apparent by day 3 for both organ constructs in the 100.0 µg/mL dose (results not shown). For lumefantrine-treated systems (**Figure S3**), no adverse effects were observed via phase imaging. Endpoint viability assays determined there were no significant declines in viability. For artesunate-treated systems (**Figure S4**), phase imaging did not depict any decline in health or variation in cell morphology. Endpoint viability assays confirmed there were no significant declines in cell viability.


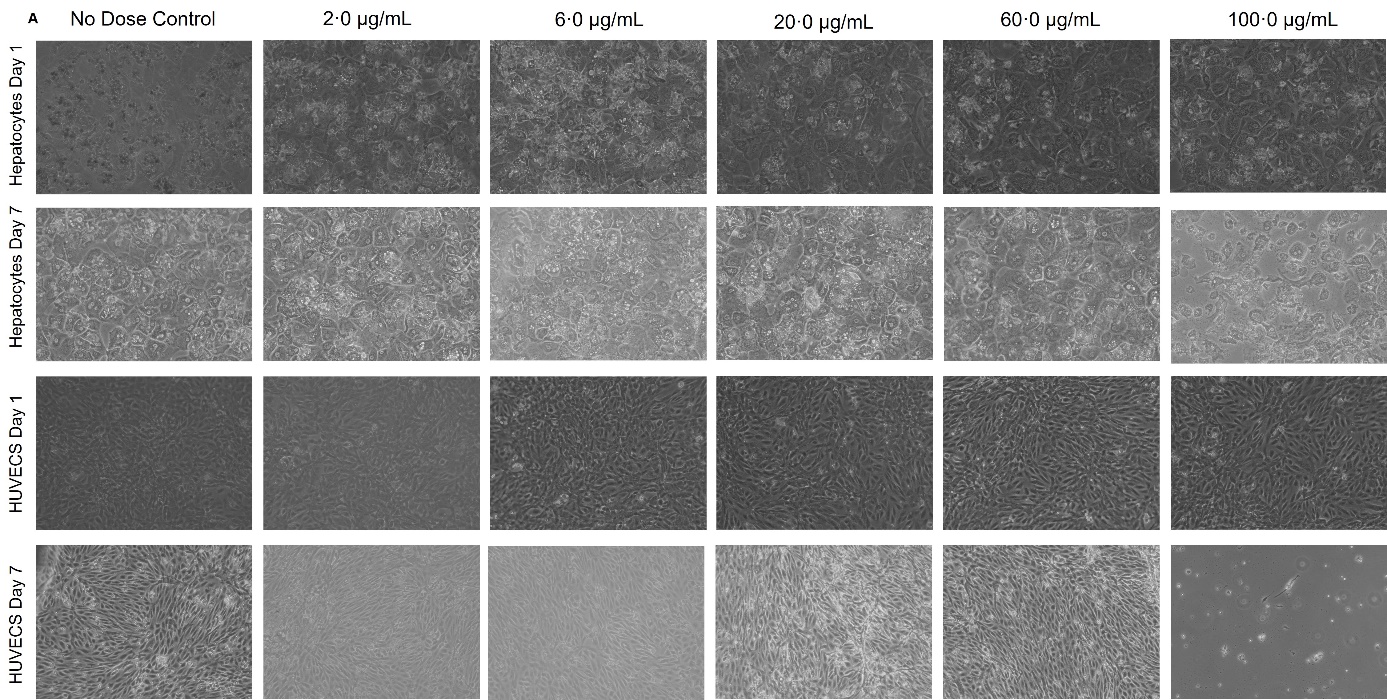


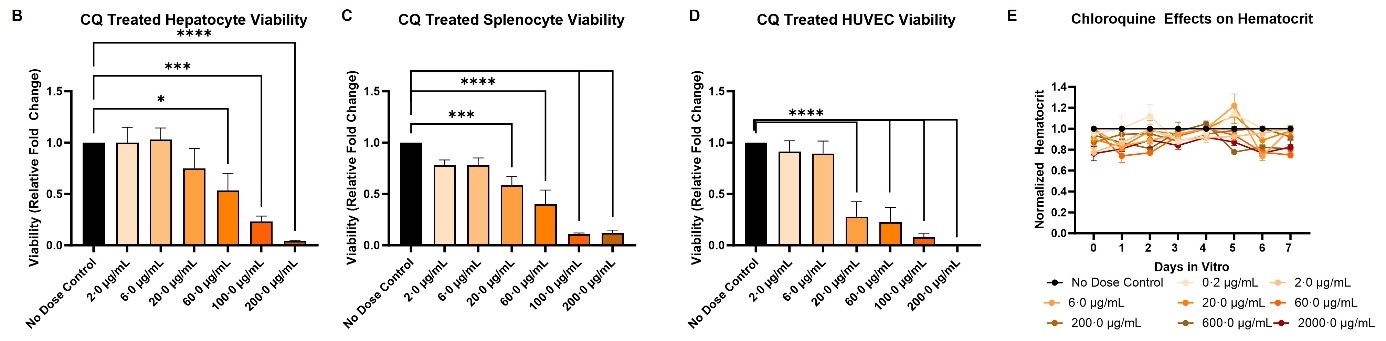


**Figure S2. Chloroquine Effects on Organ Viability.** Multiorgan systems were assembled with hepatocytes, splenocytes, HUVECs, and healthy RBCs, then dosed with various concentrations of chloroquine to monitor effects on organ construct viability. (A) Phase imaging showed increased cell death after a 100.0 µg/mL dose in both liver and endothelial organ constructs. (B-D) Viability studies were conducted for the liver, spleen, and endothelium on day 7, following the disassembly of the multi-organ systems. A dose-dependent decline in viability was observed with significant decreases in viability beginning with the 20.0 µg/mL dose for the spleen and endothelium and beginning with the 60.0 µg/mL dose for the liver. (E) Blood samples were collected daily from each system in RBC populations for each dose concentration, with no hemolytic effects observed. *p≤ 0.05, **p≤ 0.01, ***p≤ 0.001, ****p≤ 0.0001; Data was analyzed via One-way ANOVA; Mean ± SEM.

**
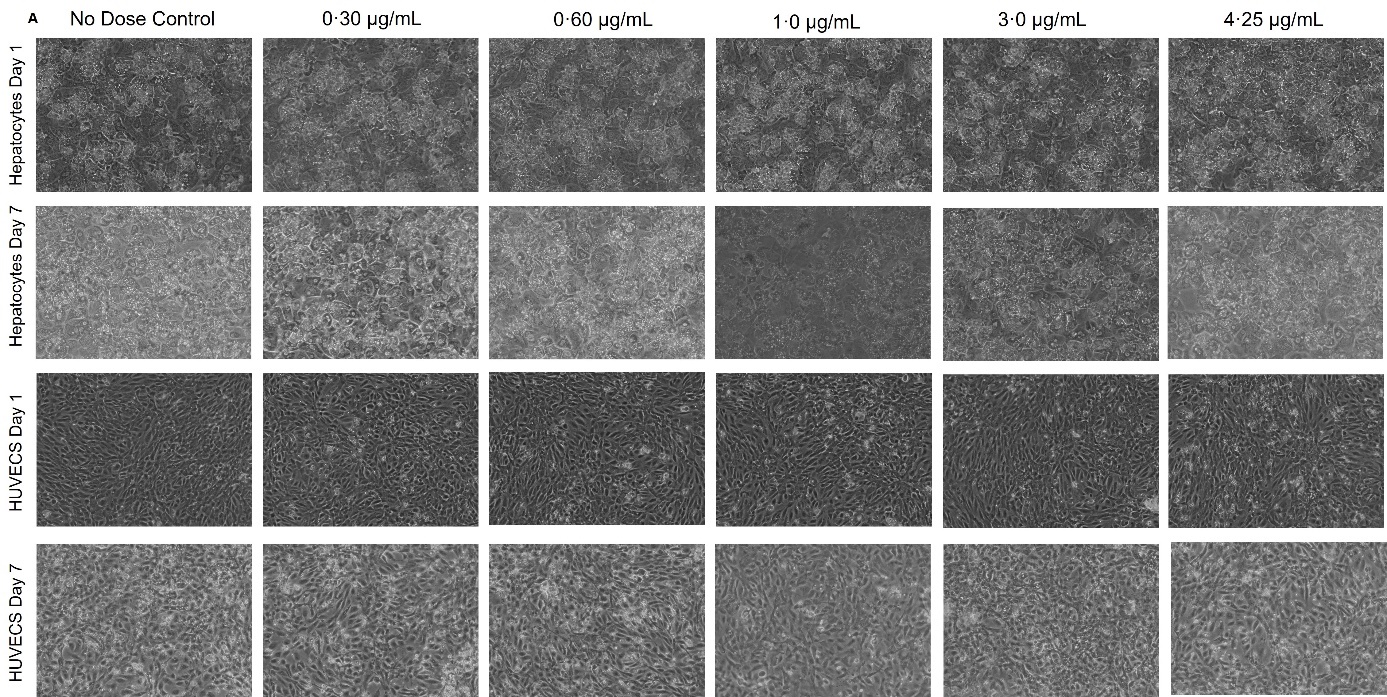
**

**
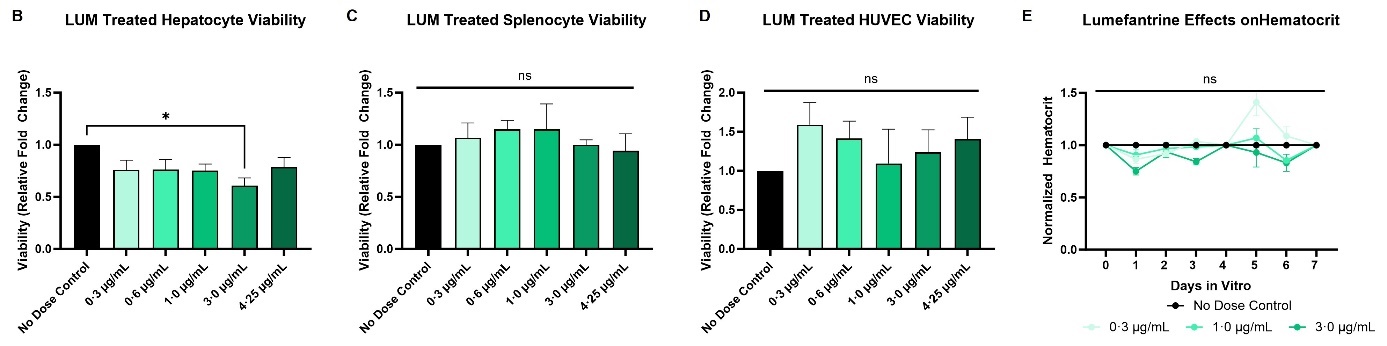
**

**Figure S3. Lumefantrine Effects on Organ Viability.** Multiorgan systems were assembled with hepatocytes, splenocytes, HUVECs, and healthy RBCs, then dosed with various concentrations of lumefantrine to monitor effects on organ construct viability. (A) Phase imaging showed no significant changes over 7 days in the liver and endothelium organ constructs. (B-D) Viability studies were conducted on day 7 following system disassembly and showed no significant decreases in liver, spleen, or endothelium viability between dose concentrations. (E) Blood samples were collected daily from each system in RBC-only populations for each dose concentration. Data was analyzed via One-way ANOVA; Mean ± SEM; No significant differences were observed.

**
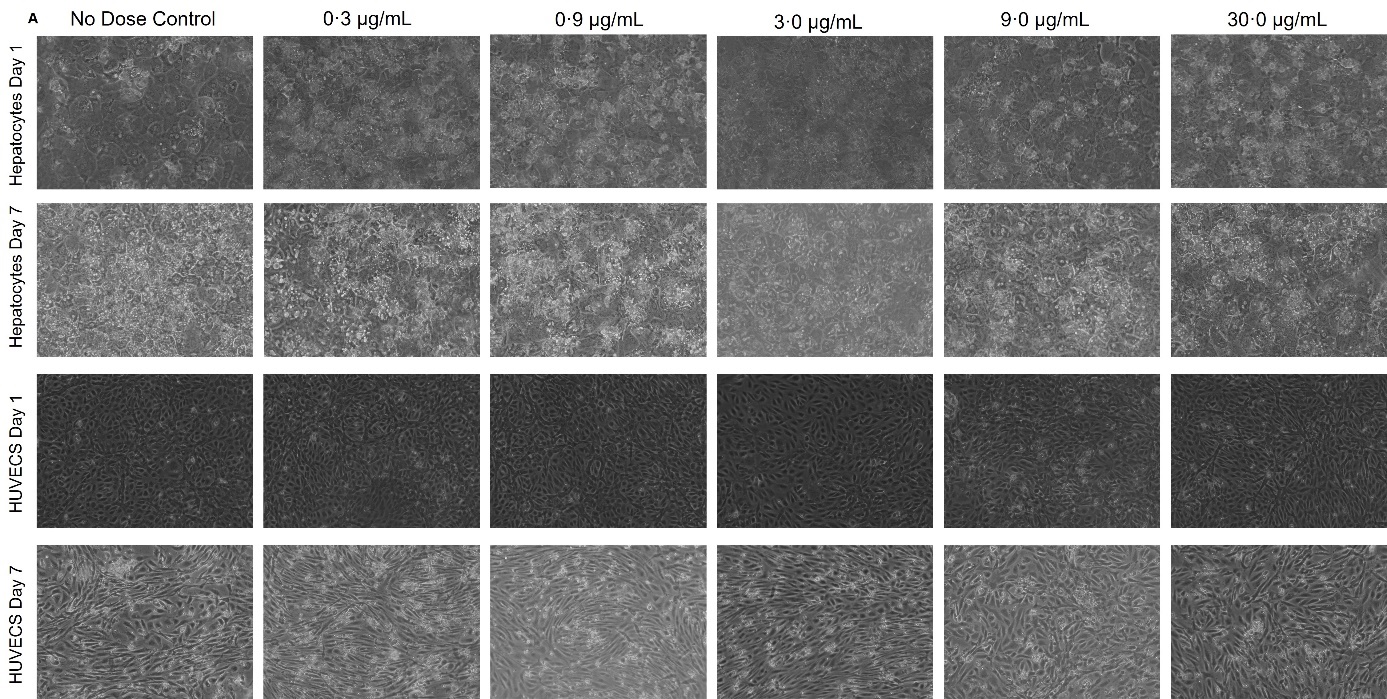
**

**
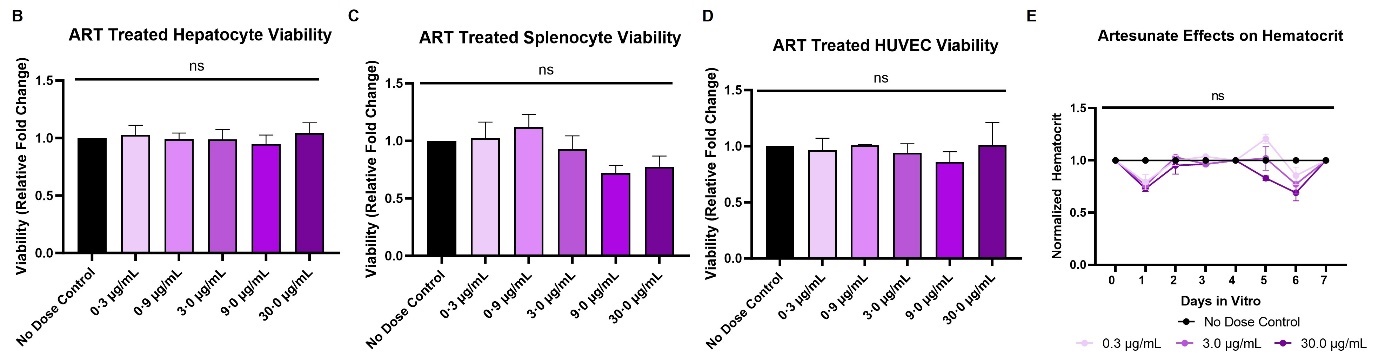
**

**Figure S4. Artesunate Effects on Organ Viability.** Multiorgan systems were assembled with hepatocytes, splenocytes, HUVECs, and healthy RBCs, then dosed with various concentrations of artesunate to monitor effects on organ construct viability. (A) Phase imaging showed no significant changes over 7 days in both liver and endothelium organ constructs. (B-D) Viability studies were conducted on day 7 following system disassembly and showed no significant decreases in liver, spleen, or endothelium viability between dose concentrations. (E) Blood samples were collected daily from each system in RBC populations for each dose concentration. Data was analyzed via One-way ANOVA; Mean ± SEM; No significant differences were observed.

**Characterization of the Malaria-on-a-Chip Phenotypic Disease Model**

Systems which contain the standard 3% hematocrit obstruct any potential for observational imaging due to the high density of erythrocytes (**Figure S5**).

**
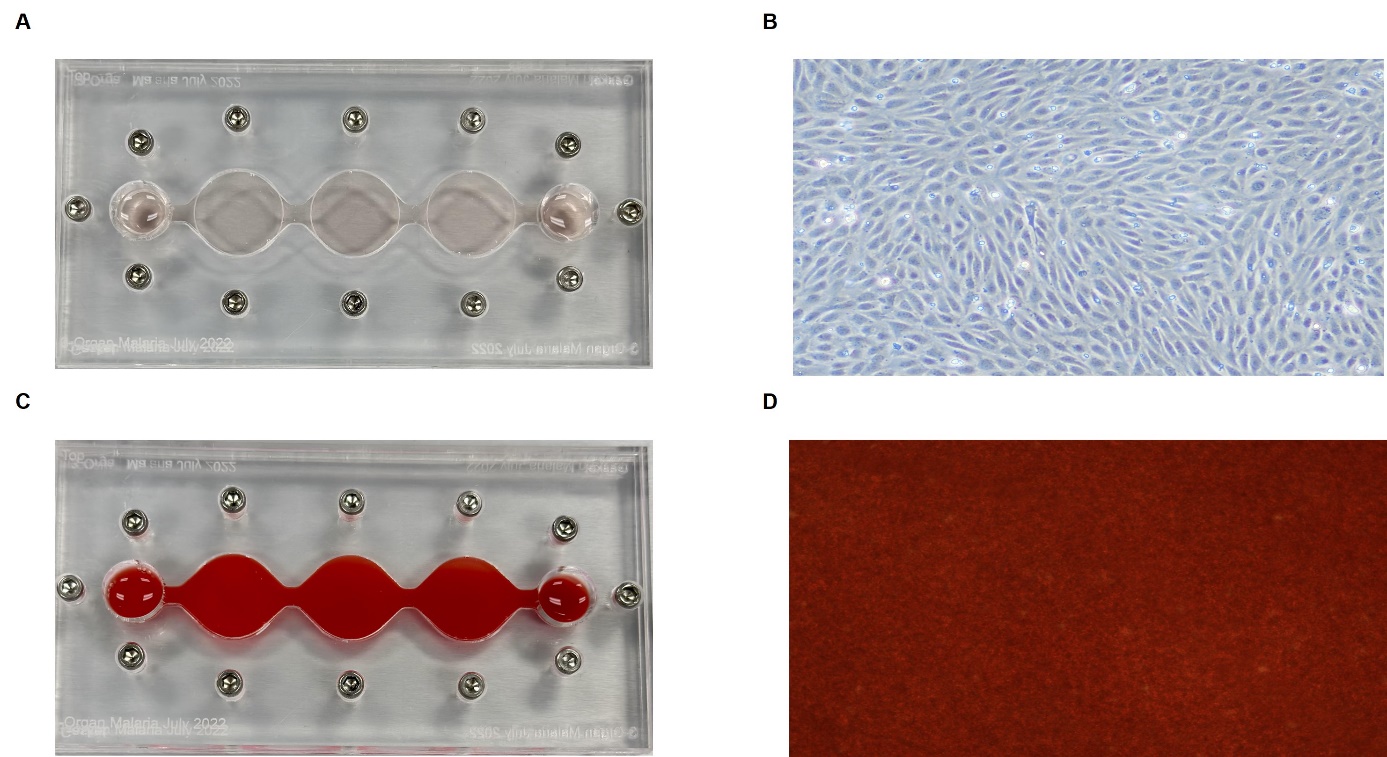
**

**Figure S5. Effects of Hematocrit Levels on Phase Imaging.** For the Malaria-on-a-Chip model, the hematocrit concentration of 3% was derived from the traditional in vitro culture of P. falciparum found in literature. An example of a system containing (A) no erythrocytes and a phase image of the (B) endothelial organ construct is shown. The addition of 3% hematocrit to the system (C) greatly reduces the ability to visually monitor the organ constructs contained within the system (D).

**Parasite Quantification via Flow Cytometry**

Scientists have classically quantified parasitemia, which is the number of iRBCs, via staining with a Giemsa Wright stain. RBCs will stain a bright pinkish color while the erythrocytic parasites will stain dark purple. This allows for both the quantification of parasitic RBCs to healthy, non-infected RBCs, as well as identification of parasite stages. While this is considered the gold standard of plasmodium quantification, it is incredibly labor intensive and subjected to human error. Flow cytometry has previously been explored in parasite quantification and as such was utilized in this study to optimize and streamline the process.

Continuous culture flasks were maintained for 33 days. Every odd day a sample was collected and used for either a thin blood smear for Giemsa staining or collected in a 96-well plate for SYBR Green I nucleic acid stain for quantification via flow cytometry. For the Giemsa stain, an infected thin blood smear was observed under a microscope (**Figure S6A**) and cells were counted to determine the ratio of infected RBCs (iRBCs) versus healthy RBCs. Samples stained with SYBR Green were processed via flow cytometry, and gating of the populations was used to determine the parasitemia levels (**Figure S6B**). Twelve flasks were maintained for each strain and the values for methods of quantification were plotted (**Figure S6C,D**) and it was determined that there were no significant differences between the 2 techniques. Flow cytometry was further utilized to monitor the replication rate of the parasite and more precisely monitor the distribution of the parasite stages that make up the total parasitemia (**Figure S7**).


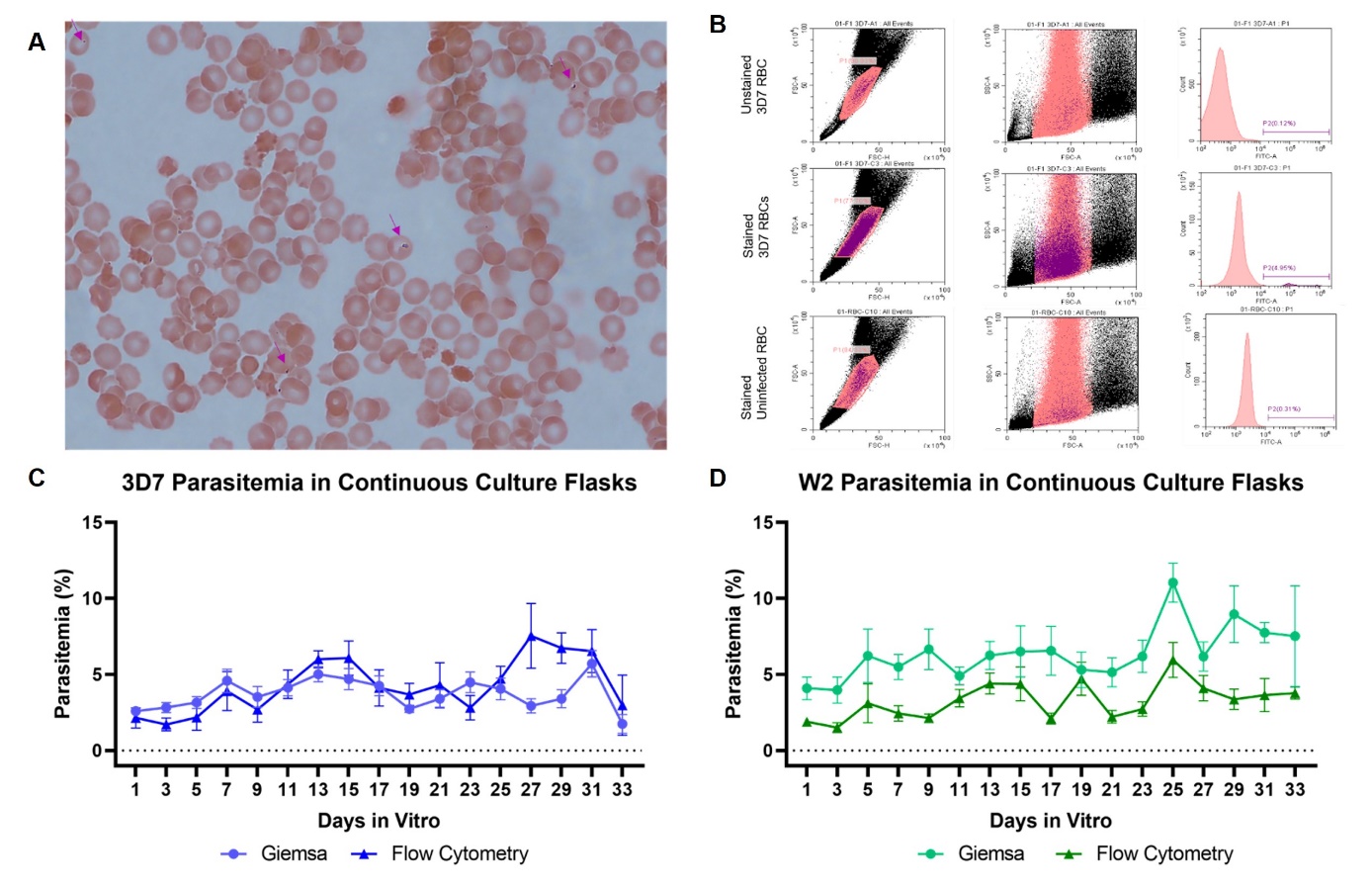


**Figure S6. Parasitemia Quantification.** (A) Image of a thin blood smear collected from a Plasmodium falciparum infected culture. (B) Gating strategy for quantification of parasites via flow cytometry. Twelve continuous culture flasks containing the (C) 3D7 strain and twelve additional flasks containing the (D) W2 strain were maintained for 33 days. Every two days, blood samples were collected and simultaneously quantified by Giemsa staining of thin blood smears and flow cytometry with SYBR Green nucleic acid stain. Data was analyzed via Two-way ANOVA; Mean ± SEM; No significant differences were observed.

**
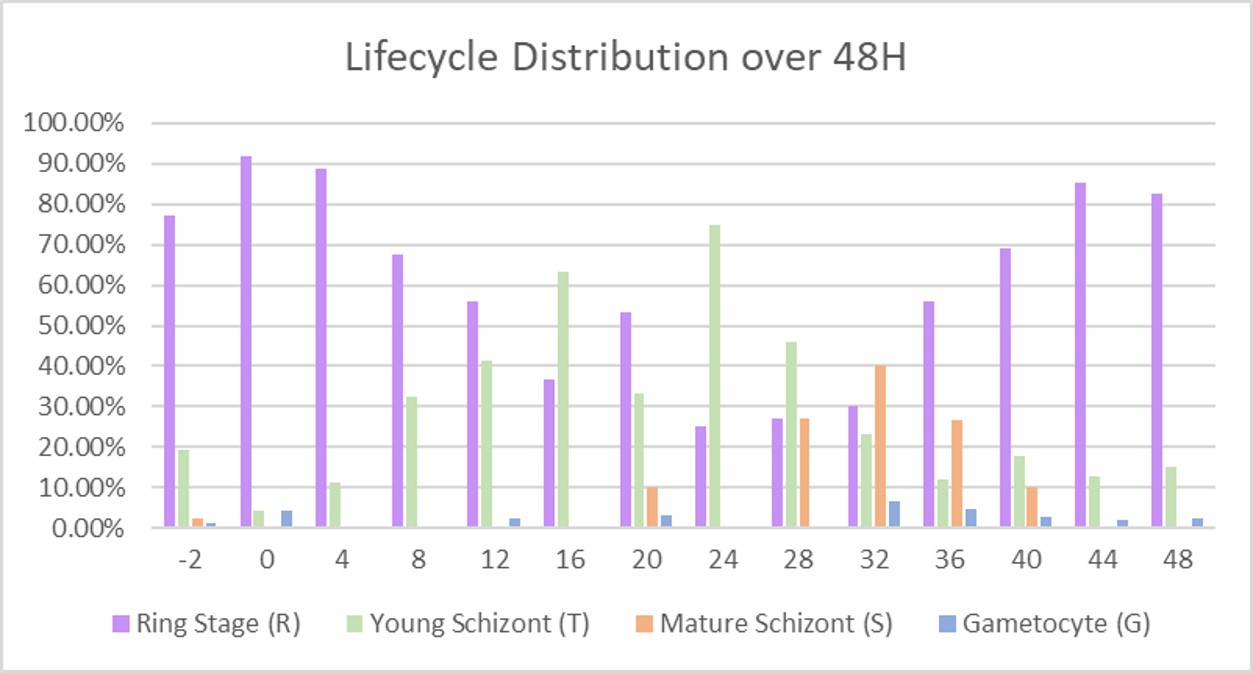
**

**Figure S7. Parasite lifecycle monitoring in the Malaria-on-a-Chip model.** P. falciparum cultures were synchronized to the ring stage prior to infecting healthy systems. Following infection, blood samples were collected at 4-hour increments to identify the percentage of each parasite stage that contributed to the total parasitemia.

**Ab/Adsorption of Antimalarial Compounds in the Malaria-on-a-Chip Model**

For the Malaria-on-a-Chip model, PDMS gaskets are sandwiched in between acrylic housing material, reducing the amount of PDMS relative to most microfluidic organ-on-a-chip devices. These gaskets provide a liquid-tight seal to define the cell culture compartments and maintain the culture system, but also absorb and subsequently absorb compounds, especially lipophilic compounds. This absorption can skew the administration of therapeutic compounds and as such, it is necessary to first determine the absorption of compounds within acellular systems before delivering a dose to multi-organ systems. Compounds were prepared at specific dose concentrations, delivered in a single bolus dose to the acellular system, and 50 µL samples were collected. As full mixing is observed within this platform by 24 hours the first samples were collected at 4-, 8-, 12-, and 24-hour time points. To monitor the concentration of the compounds over the entire experiment, samples were also collected on days 2, 3, 4, 5, 6, and 7. These samples were then quantified via HPLC-LCMS.

For chloroquine, the results of these experiments determined that the approximate absorption values for Chloroquine were dose dependent. In the first 24 hours a 72% reduction in concentration was observed for the 2.0 and 6.0µg/mL dose, 61% for the 20.0µg/mL, 29% for the 60.0µg/mL dose, 12% for the 100.0µg/mL, and 18% for the 200.0µg/mL dose. The maximum concentration (C_max_) for the doses within the platform was 1.02, 2.42, 11.82, 52.4, 90.46, 163.03µg/mL, respectively.

For lumefantrine, it was determined that approximately 78% of the compound was absorbed for the lowest dose of 0.3µg/mL, 89% for 0.6µg/mL, 85% for 1.0µg/mL, 65% for 3.0µg/mL, and 52% for the 4.25µg/mL doses. The C_max_ for the doses within the platform was 0.13, 0.24, 0.43, 1.04, and 2.04µg/mL, respectively.

For artesunate, concentrations following 24 hours of culture were drastically reduced by more than 90%. This is likely attributed to both absorption and the rapid half-life of the compound. The 0.03µg/mL dose was reduced below a detectable limit, 0.3µg/mL by 97%, 3.0µg/mL by 94%, 30.0µg/mL by 95%, and 90.0µg/mL by 93%. The C_max_ for the doses within the platform (excluding the 0.03µg/mL dose as the concentration fell below the limit of detection) was 0.04, 0.69, 6.38, and 19.29µg/mL, respectively.

**Antimalarial Off-Target Toxicity in the malaria-on-a-chip System**

As most of the chloroquine concentrations used in this study maintained supraphysiological concentrations over the course of 7 days, doses above the 6.0µg/mL dose were not expected to contribute to organ viability as it cleared the parasite. For all organ constructs exposed to either strain of infection, dramatic declines in viability were observed when compared to the infected yet untreated systems (**Figure S8**). Hepatocyte and HUVEC viability both declined significantly in each strain with chloroquine treatment of 20.0µg/mL or higher. The only variation existed with the spleen organ construct. The W2-infected spleens had a significant decline in viability with the 20.0µg/mL dose, however, the 3D7-infected spleen viability began to decline at a higher dose of 60.0µg/mL. Interestingly, a significant improvement in 3D7-infected spleen viability was observed with the 0.2µg/mL dose. Any treatment of 200.0µg/mL or higher proved to be extremely toxic to the organ constructs.

No significant deviations in organ viability were observed with any dose concentration of Lumefantrine used for either strain of the parasite (**Figure S9**). While not significant, there were notable improvements in HUVEC viability when treated with lumefantrine.

Artesunate treatment proved to be not only effective in clearing the parasite, but it also had ameliorative effects on the infected organ constructs (**Figure S10**). No significant decrease in organ viability was observed in the 3D7-infected systems. In fact, significant increases in both the liver and endothelial organ construct viabilities were observed. While the 3.0µg/mL treatment was effective in clearing the parasite considering only one bolus dose was delivered, the 30.0µg/mL dose yielded the most desirable effect within this model, clearing significantly more parasites while also benefiting the organ construct viability. For the W2-infected systems receiving artesunate treatment, the only significant decrease in viability was seen in the highest dose of 150.0µg/mL in the spleen. Otherwise, this treatment was effective in clearing the parasite without negatively impacting the organ constructs.

**
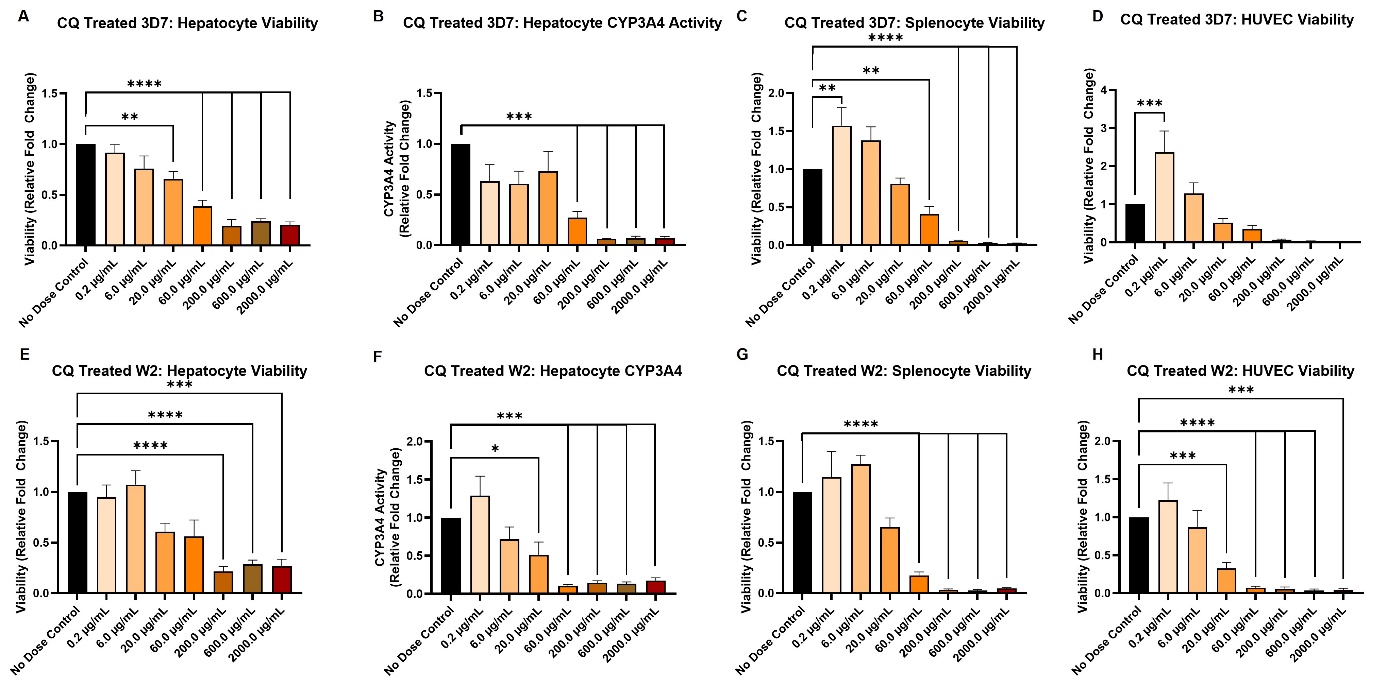
**

**Figure S8. Chloroquine Effects on Organ Constructs of the Malaria-on-a-Chip Model.** 7 Days post treatment with chloroquine, the Malaria-on-a-Chip systems were disassembled, and organ construct viability was assessed. (A-D) For the 3D7-infected systems, a dose-dependent effect on organ viability was observed. Significant declines in both (a) hepatocyte and (d) HUVEC viability was observed in treatments of 20 ·0 µg/mL and higher, while (b) hepatic function began to significantly decline with treatments of 60 ·0 µg/mL and higher. For the (c) spleen, significant declines were observed with treatments of 60 ·0 µg/mL and higher, but a significant increase in viability was observed with the 0 ·2 µg/mL dose. (E-H) Similar trends were observed in the W2-infected groups with few variations. (e-h). *p≤ 0.05, **p≤ 0.01, ***p≤ 0.001, ****p≤ 0.0001; Data was analyzed via One-way ANOVA; Mean ± SEM.

**
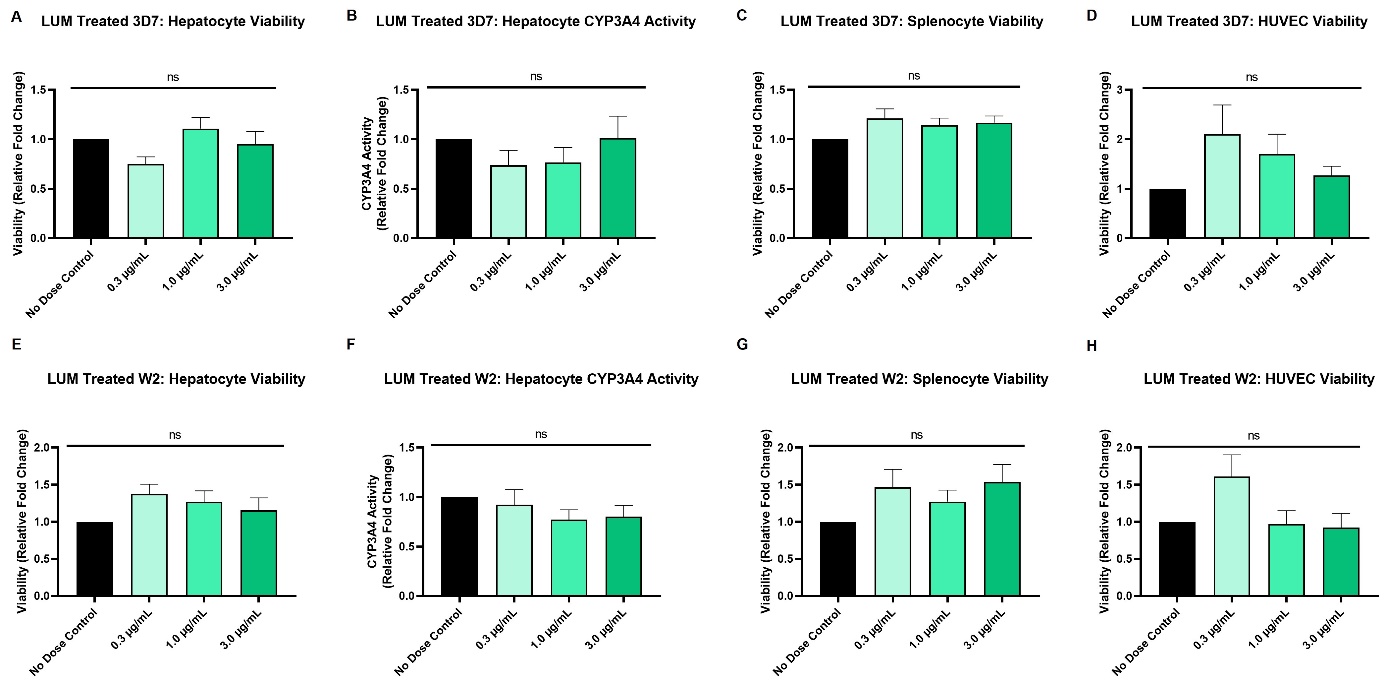
**

**Figure S9. Lumefantrine Effects on Organ Constructs of the Malaria-on-a-Chip Model.** 7 days post-treatment with lumefantrine, the Malaria-on-a-Chip systems were disassembled, and organ construct viability was assessed. No significant variations in organ construct viability were observed for any of the doses across either strain. *p≤ 0.05, **p≤ 0.01, ***p≤ 0.001, ****p≤ 0.0001 Data was analyzed via One-way ANOVA; n=12; Mean ± SEM.

**
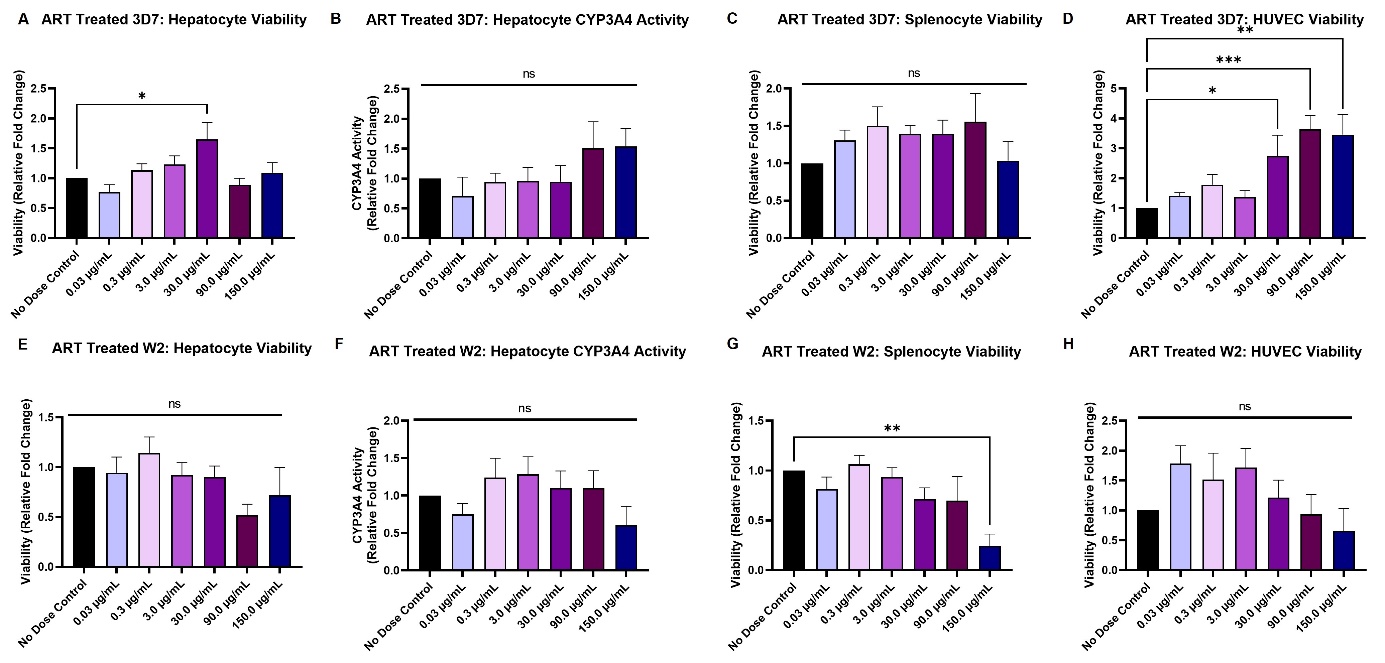
**

**Figure S10. Artesunate Effects on Organ Constructs of the Malaria-on-a-Chip Model.** 7 days post-treatment with artesunate, the Malaria-on-a-Chip systems were disassembled, and organ construct viability was assessed. (A-D) For the 3D7-infected systems, a significant increase in (A) hepatocyte viability was observed at the 30.0 µg/mL dose with no significant variations in (B) hepatic function. (C) No significant changes were observed in spleen viability while significant increases in (D) HUVEC viability were observed in treatments of 30.0 µg/mL and higher. (E-H) For the W2-infected groups, no significant changes were observed in the (E) hepatic viability, (F) hepatic function, or (H) HUVEC viability. However, a significant decrease in (G) spleen viability was observed in the highest dose of 150.0 µg/mL. *p≤ 0.05, **p≤ 0.01, ***p≤ 0.001, ****p≤ 0.0001 Data was analyzed via One-way ANOVA; Mean ± SEM.

**Pharmacokinetic (PK) – Pharmacodynamic (PD) modeling:**

Pharmacokinetic-pharmacodynamic modeling was performed in Phoenix WinNonlin 8.3 (Certara, Princeton, NJ). PK modeling of the Malaria-on-a-Chip system was performed by simultaneously fitting HPLC-MS measured time-dependent concentrations of compounds within the system across dosages to a PK model including mixing due to the recirculating behavior of the medium, ab/adsorption to the housing materials, and changes in concentration due to medium changes (**Figure S11**). The mixing parameter, defined by convective mass transport induced by the rocker-based recirculation of medium was fixed as a shared parameter among all compounds and dosages. Because each compound ab/adsorbs differently to the housing materials due to chemical characteristics, this component was fit separately for each compound, with the nonlinearity due to concentration fit to a log-linear curve. The resulting PK model to determine continuous concentration profiles based on dose was applied within Phoenix for PK/PD modeling.

PK/PD modeling for the Malaria-on-a-Chip system followed a modified sigmoidal E_max_ model developed for anti-infectives against bacterial strains from *in vitro* models.^30^ This model includes components for saturation parasitemia (N_max_), growth rate constant k_s_, maximum kill rate k_max_, 50% effective concentration (EC_50_) and Hill coefficient (h) describing sigmoidal relationship for the kill rate with respect to concentration, and a coefficient for the delay between in the compound’s effect on the parasite (dk). The rate of change in parasite number (or parasitemia), (N) with respect to the instantaneous concentration of compound (C_obs_) is given in **Equation S1**.

$$\frac{dN}{dt}=\left( k_{s}\cdot\left( 1-\frac{N}{N_{max}} \right)-\frac{k_{max}\cdot C_{obs}^{h}}{{EC}_{50}^{h}+C_{obs}^{h}}\cdot\left( 1-e^{-dk\cdot t} \right) \right)\cdot N$$

**Equation S1**

The PK and PK/PD relationships for the Malaria-on-a-Chip device were modeled using a population approach in Phoenix using each Malaria-on-a-Chip device as a population member to capture the mean, interchip and residual variance parameters using a maximum likelihood approach (**Figure S12-14**). The model was developed by simultaneously fitting the model in **Equation S1** to the raw values of parasitemia in each system with the PK profile expected for that system. The model performance was evaluated using basic goodness-of-fit plots, visual predictive checks and checking convergence of the utilized FOCE scheme.

The *in vivo* PK profiles for each compound was created from published literature in which dosing, resulting *in vivo* PK was determined, and a PK model was constructed. The model for chloroquine was adapted from the model developed by Abd-Rahman, et al 2020, in which the PK profile for chloroquine in adult humans was modeled by a two-compartment model with first-order absorption and elimination.^31^ That work was the first published study using a semi mechanistic population PK/PD model to characterize the PK/PD relationship between chloroquine plasma and whole blood concentrations and P. vivax clearance. The model for artesunate was adapted from the model developed by Li, et al 2009, in which pharmacokinetics for the artesunate and DHA (the active metabolite) were modeled using a two-compartment model with rapid initial distribution phase. ^32^ As both the artesunate and DHA contribute to the efficacy of the artesunate administration, though with different potencies, the DHA concentrations were converted to an equivalent artesunate concentration, adjusting for differences in EC_50_, to produce a combined equivalent concentration to be used in the PK/PD translation model. The *in vivo* PK model for lumefantrine was adapted from the one-compartment model provided in Hietala et al 2010.^33^ To determine translational predictive value of the Malaria-on-a-Chip with PK/PD modeling, the model relationship with parameters determined from the Malaria-on-a-Chip PK/PD modeling was treated as a putative PK/PD relationship for *in vivo* response. Recommended dosing regimens from the CDC “Malaria in the United States: Treatment Tables” (2023) were supplied to the *in vivo* pharmacokinetic models in Phoenix adapted from the literature models to determine the predicted PK profile *in vivo*.^34^

In the cases of artesunate and lumefantrine, the recommended combination dosing using both compounds was separated such that each drug was applied individually at the dosing used in the combination regimen without the co-administered compound. Following the prediction of the *in vivo* PK, the Malaria-on-a-Chip PK/PD relationships were applied to determine the translationally predicted effect of these dosages on the *P. falciparum* parasite.

The dosing regimens applied to the model for a 60 kg adult, are as follows: chloroquine phosphate dosed at 1000 mg, followed by 500 mg at 6, 24, and 48 hr; artesunate dosed at 120 mg (2 mg/kg) daily for 7 days; and lumefantrine dosed at 480 mg at 0, 8, 24, 36, 48, 60. For comparison, the PK/PD model was also used to predict the outcome using only the first dose of the dosing regimen, such that a single 1000 mg dose of chloroquine phosphate, 120 mg dose of artesunate, or 480 mg dose of lumefantrine was modeled alongside the multi-dose regimen.

To mimic the *in vivo* replication rate for *in vitro* to *in vivo* extrapolation, the *P. falciparum* replication rate *in vivo* was determined from values presented in White 2017, indicating a mean increase in parasite number from 10^4^ to 10^8^ parasites over 6-8 days, using 10^8^ parasites as an asymptotic parasite number.^35^

To translate the maximum kill rate between the Malaria-on-a-Chip system, the maximum kill rate, k_max_, was scaled according to the relative metabolic activities between *in vivo* and the Malaria-on-a-Chip system, using the initial doubling rate as a proxy and utilizing an exponential growth model. Over the first asexual cycle *in vivo*, parasites increase by a factor of 10 over approximately 2 days, whereas in the Malaria-on-a-Chip system the parasites double every 3 days. The calculated replication rate *in vivo* of 0.048 hr^-1^ and replication rate in the Malaria-on-a-Chip system of 0.0096 hr^-1^ resulted in a kill rate scaling factor of 5.0 to translate a given compound’s PK/PD k_max_ calculated in the Malaria-on-a-Chip to a predicted k_max_ *in vivo*.

A digital twin is a digital representation of a physical system (the corresponding physical twin) that mimics the structure, context, and behavior of the physical system, created and updated with data from the physical system such that the digital twin has predictive value and can inform decisions. A bidirectional interaction between the virtual and the physical reinforces the utilization and applicability of the digital twin. Digital medical twins apply the digital twin approach to medical applications, combining models of human biology with operational data to create digital representations of patients or devices to make predictions and optimize therapy. The PK/PD model provides digital medical twins for the for the Malaria-on-a-Chip devices, which can support creating digital medical twins for patients or populations.


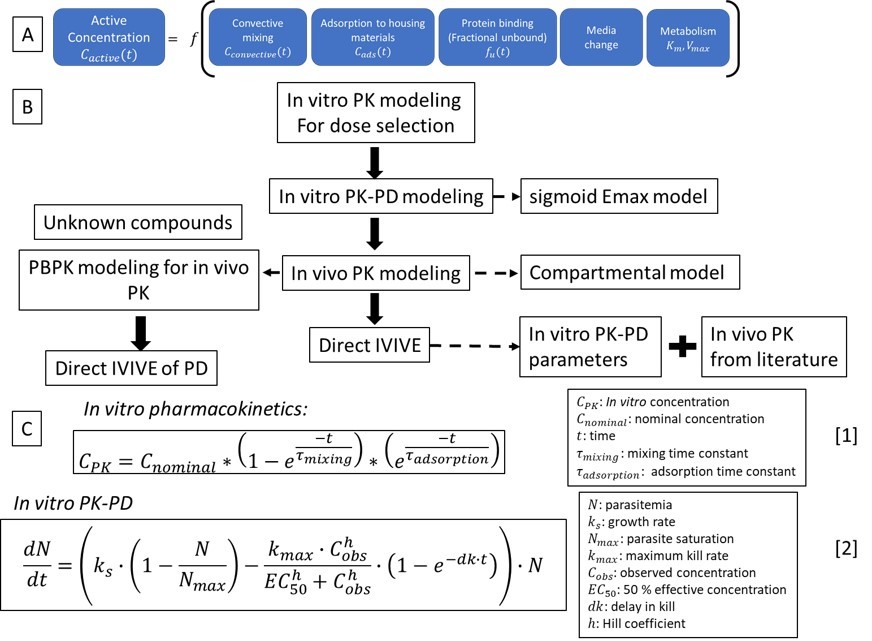


**Figure S11. PK/PD modeling workflow and equations.** (A) Factors contributing to the active in vitro concentration on the Malaria-on-a-Chip platform (B) Modeling workflow for the translational paradigm includes in vitro PK modeling which is an input to develop the in vitro PK-PD relationship. Next, in vivo PK profiles are generated using clinical literature data. For unknown compounds PBPK modeling will be an avenue to estimate a concentration profile based on the physiochemical properties of the drug. Finally, scaled in vitro parameters (for differences in growth rate) and in vivo PK profiles are used to predict parasite clearance (C) Equations used for in vitro PK model, in vitro PK-PD and in vivo PD prediction.


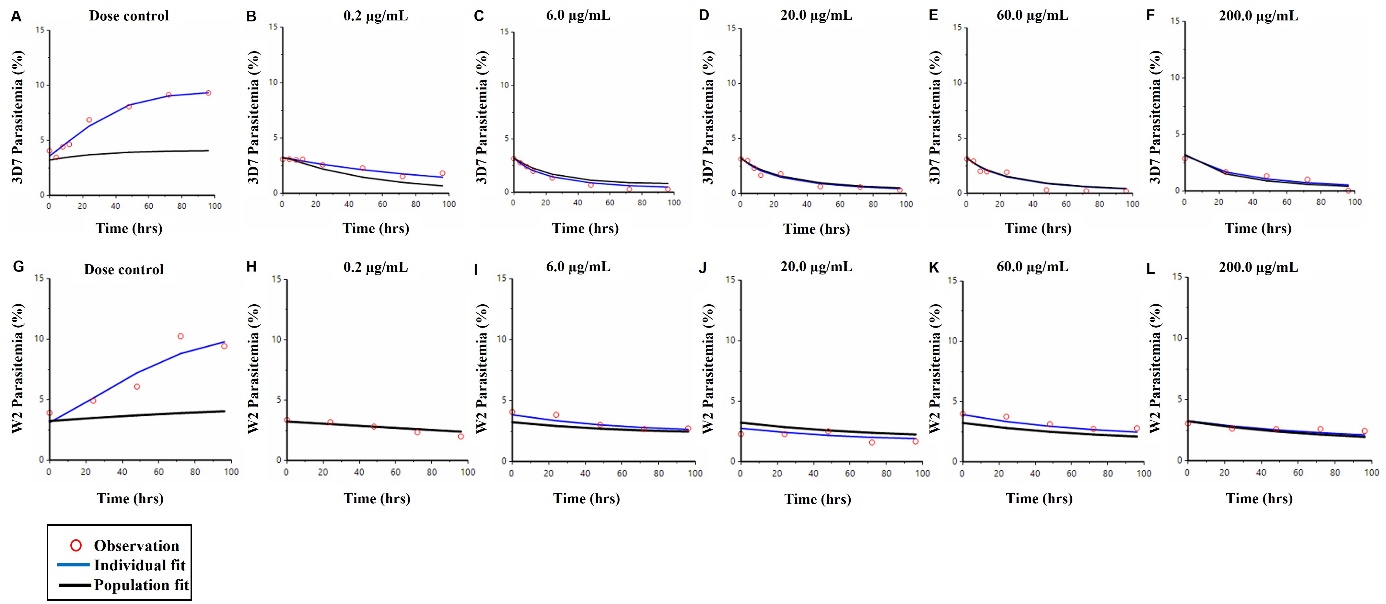


**Figure S12. Interchip variations for chloroquine treatment groups.** (A-F) Depiction of individual 3D7 system parasitemia plotted alongside the total system population parasitemia when treated with chloroquine. (G-L) Depiction of individual W2 system parasitemia plotted alongside the total system population parasitemia when treated with chloroquine.


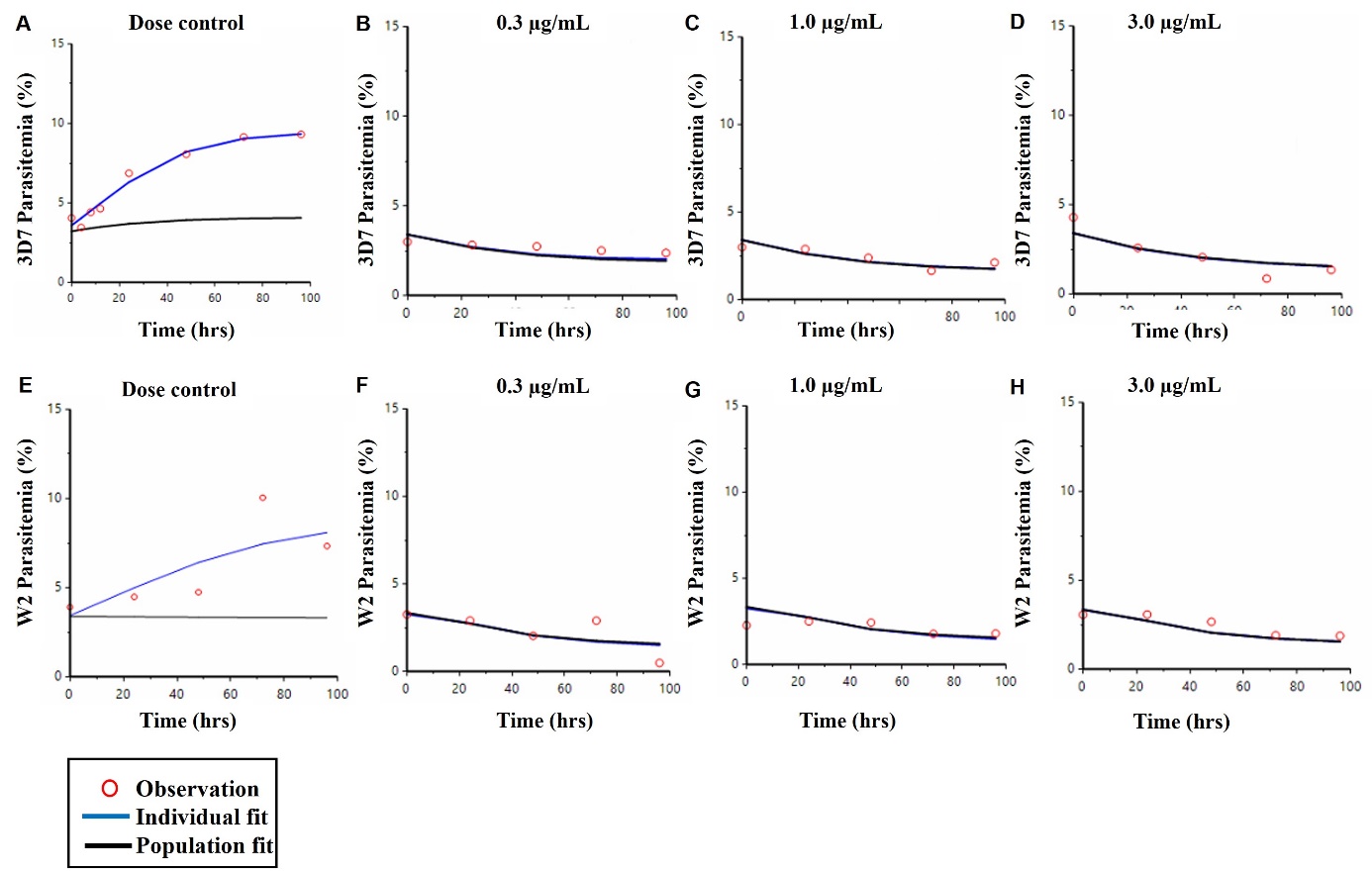


**Figure S13. Interchip variations for lumefantrine treatment groups.** (A-D) Depiction of individual 3D7 system parasitemia plotted alongside the total system population parasitemia when treated with lumefantrine. (E-H) Depiction of individual W2 system parasitemia plotted alongside the total system population parasitemia when treated with lumefantrine.


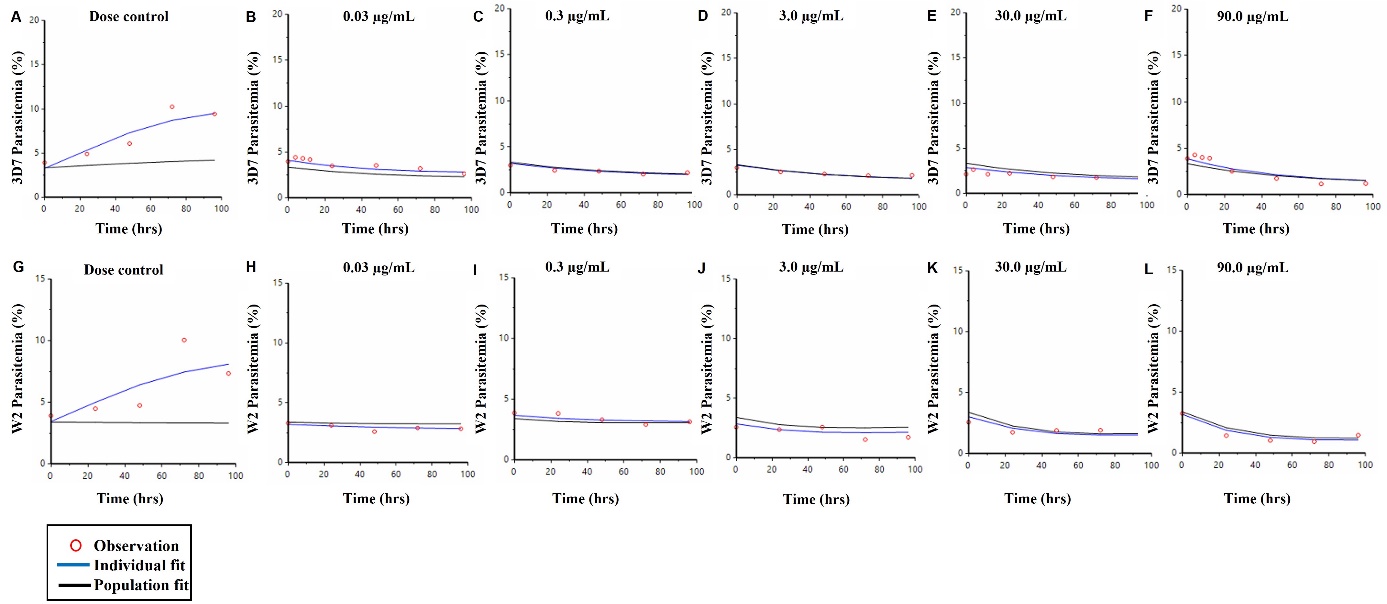


**Figure S14. Interchip variations for artesunate treatment groups.** (A-F) Depiction of individual 3D7 system parasitemia plotted alongside the total system population parasitemia when treated with artesunate. (G-L) Depiction of individual W2 system parasitemia plotted alongside the total system population parasitemia when treated with artesunate.

**Translating Pharmacokinetic and Pharmacodynamic Interactions within the malaria-on-a-chip Model to predict single-dose outcome *in vivo***

The *in vitro* to *in vivo* extrapolation PK/PD model, combining the response parameters (**Table** 1) with the predicted PK profiles (**Figure 9**) produced predicted *in vivo* responses of 3D7 and W2 strains of *P. falciparum* to single doses or recommended dosing regimens for chloroquine, artesunate, and lumefantrine. The predicted parasitemia for single doses (**Figure S15**) resulted in a reduced parasitemia of the 3D7 strain with chloroquine, while the W2 was not affected enough to prevent worsening of the infection. The artesunate single doses were ineffective for both the 3D7 and W2 strains, with an initial decrease in 3D7 parasitemia delaying parasite growth, and a shorter delay in W2 growth. The single doses of lumefantrine predicted a marked improvement in parasitemia in both the 3D7 and W2 strains, with the 3D7 nearing an 11-log reduction at day 7, and the W2 approaching a 12-log reduction in parasite at 7 days. Because both chloroquine and lumefantrine exhibit prolonged plasma concentrations lasting several days, while artesunate exhibits a very short period of elevated plasma concentration of artesunate or DHA, single dosing is expected to cause an improvement over several days of parasitemia when the strain is susceptible to either chloroquine or lumefantrine at effective concentrations, while artesunate’s effects are limited by its very short half-life.


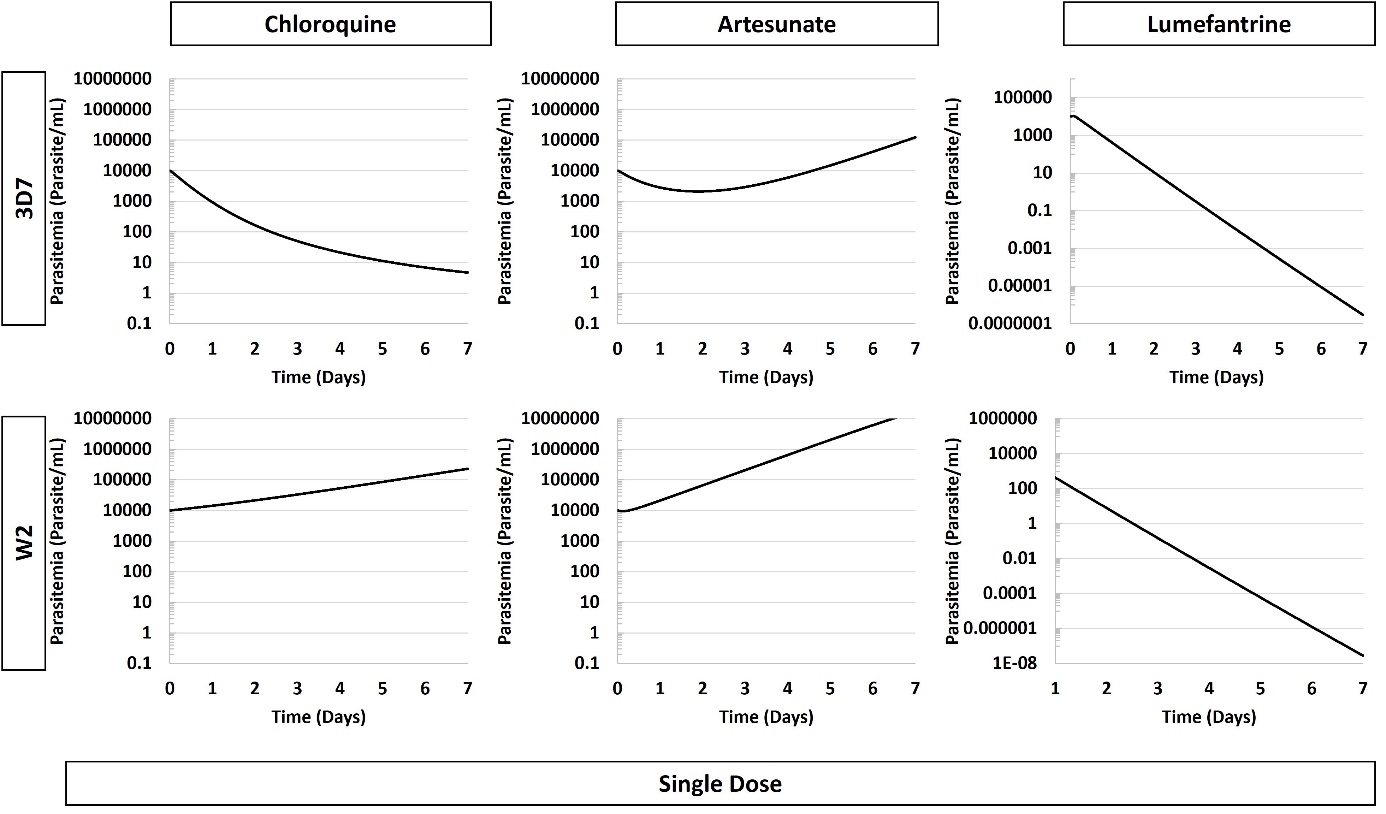


**Figure S15. Predicted in vivo parasitemia for single dosing of chloroquine (left), artesunate (middle), or lumefantrine (right) applied to infections of 3D7 (top) and W2 (bottom) *P. falciparum* strains.** An initial value of 10,000 parasites/mL was used for the onset of dosing. The model predicted pronounced strain-specific effects of chloroquine and artesunate single dosing, with a less pronounced strain-specific effect of lumefantrine.
